# Exploring the biocombinatorial potential of benzoxazoles: generation of novel caboxamycin derivatives

**DOI:** 10.1186/s12934-017-0709-6

**Published:** 2017-05-25

**Authors:** Armando A. Losada, Carmen Méndez, José A. Salas, Carlos Olano

**Affiliations:** Departamento de Biología Funcional e Instituto Universitario de Oncología del Principado de Asturias (I.U.O.P.A), Universidad de Oviedo, C/Julian Claveria s/n, 33006 Oviedo, Asturias Spain

**Keywords:** Gene expression, Heterologous expression, Nataxazole, Polyketide, Streptomyces

## Abstract

**Background:**

The biosynthesis pathway of benzoxazole compounds caboxamycin and nataxazole have been recently elucidated. Both compounds share one of their precursors, 3-hydroxyanthranilate (two units in the case of nataxazole). In addition, caboxamycin structure includes a salicylate moiety while 6-methylsalycilate is the third scaffold in nataxazole. Pathways cross-talk has been identified in caboxamycin producer *Streptomyces* sp. NTK937, between caboxamycin and enterobactin pathways, and nataxazole producer *Streptomyces* sp. Tü6176, between nataxazole and coelibactin pathways. These events represent a natural form of combinatorial biosynthesis.

**Results:**

Eleven novel caboxamycin derivatives, and five putative novel derivatives, bearing distinct substitutions in the aryl ring have been generated. These compounds were produced by heterologous expression of several caboxamycin biosynthesis genes in *Streptomyces albus* J1074 (two compounds), by combinatorial biosynthesis in *Streptomyces* sp. NTK937 expressing nataxazole iterative polyketide synthase (two compounds) and by mutasynthesis using a nonproducing mutant of *Streptomyces* sp. NTK937 (12 compounds). Some of the compounds showed improved bioactive properties in comparison with caboxamycin.

**Conclusions:**

In addition to the benzoxazoles naturally biosynthesized by the caboxamycin and nataxazole producers, a greater structural diversity can be generated by mutasynthesis and heterologous expression of benzoxazole biosynthesis genes, not only in the respective producer strains but also in non-benzoxazole producers such as *S. albus* strains. These results show that the production of a wide variety of benzoxazoles could be potentially achieved by the sole expression of *cbxBCDE* genes (or orthologs thereof), supplying an external source of salicylate-like compounds, or with the concomitant expression of other genes capable of synthesizing salicylates, such as *cbxA* or *natPK*.

**Electronic supplementary material:**

The online version of this article (doi:10.1186/s12934-017-0709-6) contains supplementary material, which is available to authorized users.

## Background

Sequencing of *Streptomyces* sp. NTK937 genome, a deep-sea sediment strain, revealed up to 35 putative gene clusters for the biosynthesis of secondary metabolites [1], including that for the biosynthesis of antibiotic caboxamycin. This compound belongs to the family of benzoxazoles [2], which includes compounds such as calcimycin [3], nataxazole [4], and A33853 [5], whose respective biosynthesis pathways have been characterized [6–9]. The benzoxazole scaffold is known to bear different pharmacological properties such as being cytotoxic and/or antibiotic [10], possessing anti-leishmanial properties [11], or inhibiting the replication of hepatitis C virus [12].

**Fig. 1:**
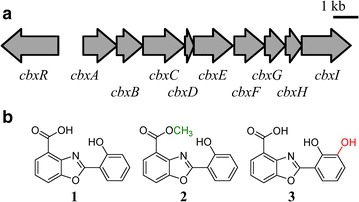
Caboxamycin biosynthesis gene cluster and chemical structure of related compounds. **a** Genetic organization of caboxamycin biosynthesis gene cluster. **b** Chemical structure of caboxamycin, **1**; *O*-methylcaboxamycin, **2**; and 3′-hydroxycaboxamycin, **3**

Recently, our group has elucidated the pathway for caboxamycin biosynthesis comprising nine structural genes, *cbxA*-*I* (Fig. 1a). Caboxamycin derives from chorismate through the generation, activation and condensation of two precursors, 3-hydroxyanthranilate (3HAA) and salicylate (SA) [13]. Five of the estructural genes, *cbxA, cbxB, cbxD* and *cbxE*, encoding a salicylate synthase, 3-oxoacyl-ACP-synthase, ACP, and amidohydrolase respectively, were found essential for caboxamycin biosynthesis. The remaining structural genes, *cbxC*, *cbxF*, *cbxG*, *cbxH* and *cbxI*, encoding a AMP-dependent synthetase-ligase, 3-deoxy-d-arabinohept-2-ulosonate-7-phosphate synthase, 2,3-dihydro-2,3-dihydroxybenzoate dehydrogenase, isochorismatase and anthranilate synthase respectively, have paralogs distributed throughout *Streptomyces* sp. NTK937 genome and thus they are not essential for the biosynthesis of caboxamycin. In addition, the biosynthesis gene cluster contains a positive SARP-like transcriptional regulator, *cbxR*. In this previous study, *Streptomyces* sp. NTK937 has been shown to naturally produce two different compounds: caboxamycin (**1**) and its methyl ester, *O*-methylcaboxamycin (**2**) (Fig. 1b), whose biosynthesis depends on an uncharacterized *O*-methyltransferase located neither within nor near the cluster. Furthermore, upon gene replacement of the salicylate synthase *cbxA*, a third compound was observed, 3′-hydroxycaboxamycin (**3**) (Fig. 1b), stemming from the cross-talk between the caboxamycin biosynthesis pathway and 2,3-dihydroxybenzoate (DHB) generated in the biosynthetic pathway for the siderophore enterobactin [13]. This phenomenon, together with our previous studies of the nataxazole biosynthetic cluster, where we identified cross-talk between the nataxazole and coelibactin biosynthesis pathways that led to biosynthesis of benzoxazole UK-1 [7, 8], sparked our interest for the generation of novel benzoxazoles exploring the biocombinatorial potential of these family of compounds.

In this work we report the generation of 16 novel caboxamycin derivatives (**4**–**19**) bearing distinct substitutions in the aryl ring have been generated. These compounds were produced by using different molecular approaches such as heterologous expression of selected genes, combinatorial biosynthesis using genes from different pathways and feeding different precursors to selected mutants by mutasynthesis.

## Results

### Heterologous expression of caboxamycin biosynthesis genes in *Streptomyces albus* J1074

**Fig. 2:**
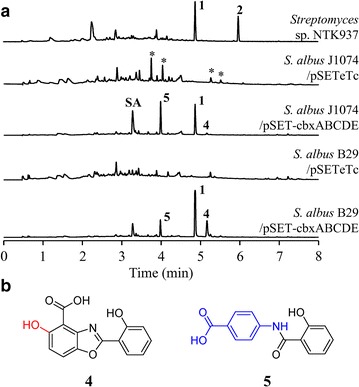
Compounds generated by heterologous expression of caboxamycin biosynthesis genes in *S. albus* J1074. **a** UPLC analysis of *Streptomyces* sp. NTK937 wild-type and *S. albus* strains J1074 and B29 harboring pSET-cbxABCDE or control plasmid pSETeTc. Chromatograms were analyzed at 330 nm. **SA** stands for salicylic acid. *Asterisks* indicate paulomycins and paulomenols. **b** Chemical structure of novel compounds **4** and **5**

The structural genes from the caboxamycin biosynthesis cluster, *cbxABCDEFGHI,* have previously been expressed as a whole in *Streptomyces lividans* JT46 as heterologous host, leading to the production of caboxamycin [13]. Since paralogs to *cbxFGHI* exist in *Streptomyces* sp. NTK937 (probably involved in primary metabolism) we assumed that a similar situation could occur in other *Streptomyces* spp. Subsequently we aimed to induce the heterologous production of caboxamycin by solely expressing the *cbxABCDE* genes, encoding a salicylate synthase, oxoacyl-ACP synthase, AMP-dependant synthetase-ligase, ACP and amidohydrolase respectively, in two *Streptomyces albus* strains: *S. albus* J1074 [14] and B29 [15]. The former produces the glycosylated antibiotic family of paulomycins that involves the participation of genes *plm15*-*18*, orthologs to *cbxFGHI* and responsible for the biosynthesis of the 3HAA-derived core of paulomycins [16]. On the other hand, in *S. albus* B29, genes *plm15*-*16*, orthologs of *cbxHI*, have been replaced by an apramycin resistance cassette, thus blocking paulomycin biosynthesis [15]. Expression of *cbxABCDE* under the control of constitutive promoter ermE*p [17] using a pSET152-derived plasmid, pSET-cbxABCDE, afforded a substantial production of **1** in both strains (Fig. 2), clearly showing the existence of orthologs for *cbxFGHI* within primary metabolism genes in the host strains. In addition, two novel products were generated (Fig. 2): compound **4**, a benzoxazole-hydroxylated version of **1**, barely detectable in the J1074 strain, but clearly produced in the B29 strain (Additional file 2: Table S1, Additional file 3: Figure S3); and **5**, a *p*-aminobenzoate (PABA) amide-linked to a salicylate moiety, present in both strains (Additional file 2: Table S2, Additional file 3: Figure S4).

### Heterologous expression of *natPK* in *Streptomyces* sp. NTK937

**Fig. 3:**
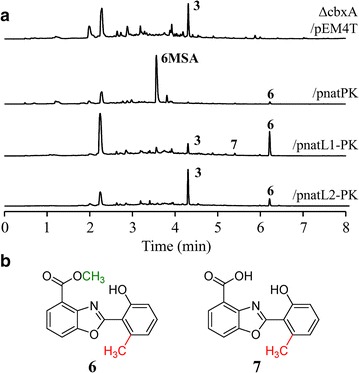
Compounds generated by combinatorial biosynthesis in *Streptomyces* sp. NTK937. **a** UPLC analysis of *Streptomyces* sp. NTK937 mutant strain ΔcbxA harboring plasmids pEM4T (control), pnatPK, pnatL1-PK or pnatL2-PK. Chromatograms were analyzed at 330 nm. **6MSA** stands for 6-methylsalicylic acid. **b** Chemical structure of novel compounds **6** and **7**

Within the scope of our research, ortholog genes from the related biosynthetic cluster of benzoxazole nataxazole [7] were heterologously expressed in several *Streptomyces* sp. NTK937 caboxamycin non-producing mutants, successfully restoring caboxamycin biosynthesis in all cases, except for the amidohydrolase coding *natAM* [13]. However, the nataxazole cluster also contains a structural gene with no ortholog in caboxamycin biosynthesis, *natPK*, an iterative type I polyketide synthase responsible for the biosynthesis of the 6-methylsalicylate (6MSA) moiety present in nataxazole. This gene was expressed, using plasmid pnatPK, in the *Streptomyces* sp. NTK937 non-producing strain ΔcbxA, thus generating noticeable quantities of the precursor 6MSA (Fig. 3). Furthermore, low levels of a caboxamycin derivative (**6**) were produced as a consequence of combinatorial biosynthesis. Compound **6** carries both the aryl methylation characteristic of 6MSA, plus the methyl esterification ascribed to the unlocated *O*-methyltransferase (Additional file 2: Table S3, Additional file 3: Figure S5). We sought to improve production of **6** using plasmid constructs bearing both *natPK* and one of the AMP-dependant synthetase-ligases from the nataxazole cluster. The combined expression of *natL1*-*natPK* (pnatL1-PK) afforded the production of highest amounts of **6**, whereas simultaneous expression of *natL2*-*natPK* (pnatL2-PK) favored instead the production of **3**.

A small peak observed in ΔcbxA/pnatL1-PK cultures, compound **7**, with a retention time of 5.4 min (Fig. 3), possibly corresponds to the acid version of **6**, given its coherently shorter than **6** (6.15 min) but higher than **1** retention time (4.90 min). This peak was consistently observed along the different experiments performed, but its drastically low levels of production prevented its isolation and spectroscopic characterization, although HPLC–MS analysis is coherent with this hypothesis, showing a mass of *m/z* 270.07 [*M*+H]^+^.

### Generation of novel derivatives by mutasynthesis

Upon observing how amenable *Streptomyces* sp. NTK937 was to the combination of similar precursors to yield novel caboxamycins, we sought to obtain novel compounds following a mutasynthesis approach, a strategy involving the addition of modified precursors in biosynthetically-blocked mutants in order to afford novel derivatives of the original product. As a host, the *Streptomyces* sp. NTK937 non-producing double mutant strain ΔcbxA/ΔentC was selected, since syntheses of both SA and DHB are abolished, thus allowing for better resource allocation towards the production of novel caboxamycins.

**Fig. 4:**
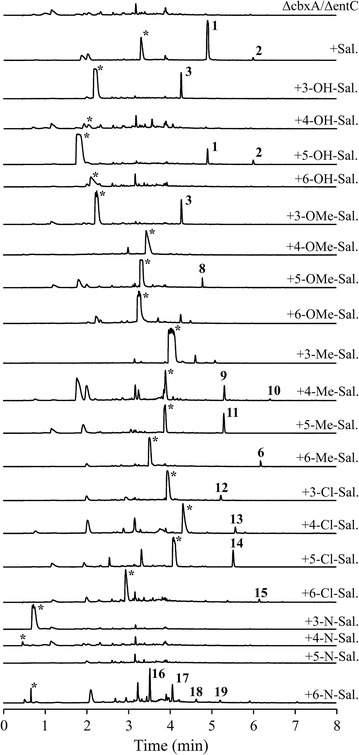
UPLC analysis of mutasynthesis assays in *Streptomyces* sp. NTK937 ΔcbxA/ΔentC. Chromatograms were analyzed at 330 nm. *Asterisks* indicate the precursor used. *Sal.* salicylate

**Fig. 5:**
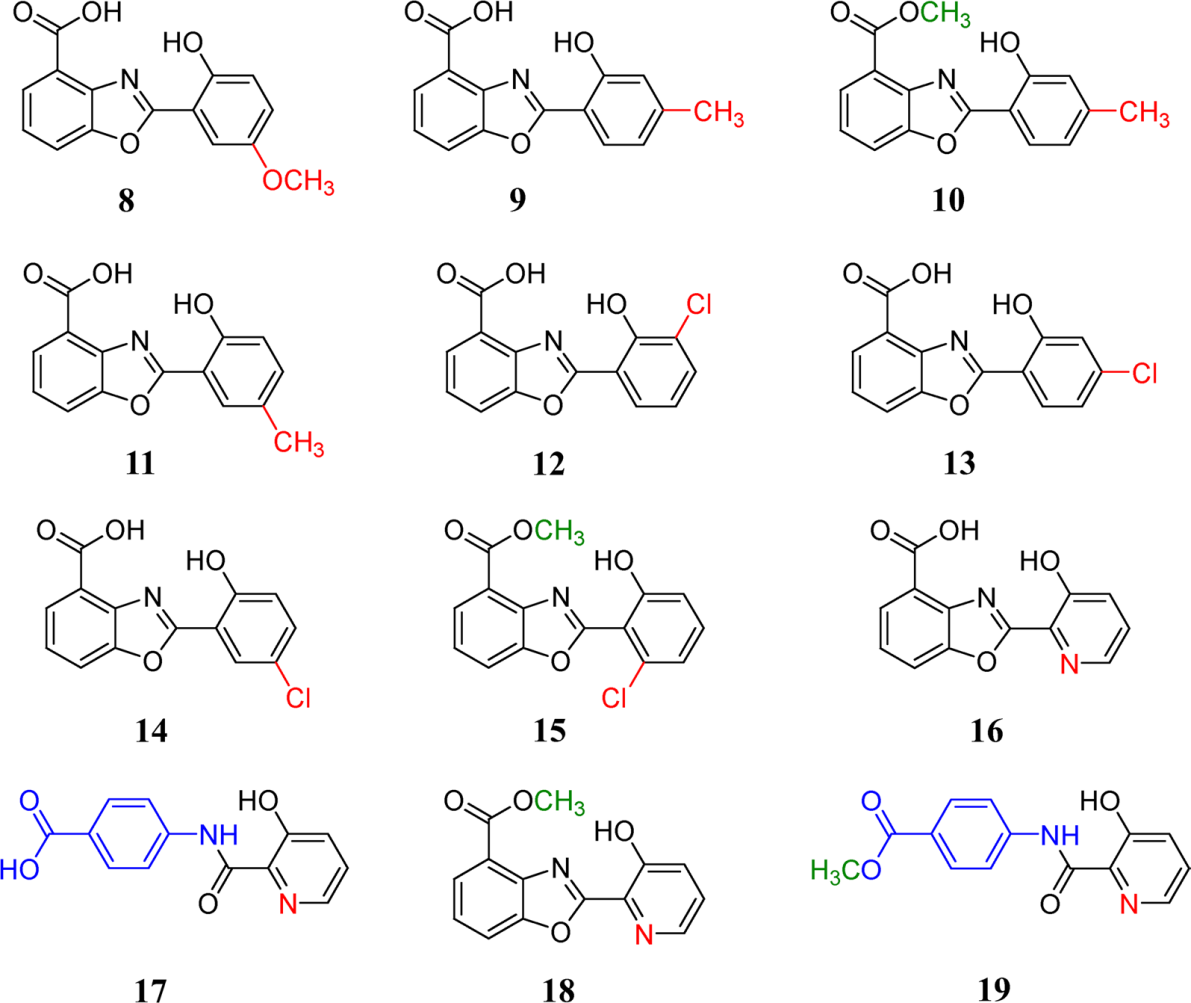
Chemical structure of compounds **8**–**19** obtained by mutasynthesis in *Streptomyces* sp. NTK937 ΔcbxA/ΔentC

A selection of different precursors, all bearing carboxylate groups attached to cyclic molecules, was initially tested and it was found that under standard working conditions, the mutant strain only assimilates molecules of salicylate structure, i.e., aromatic acids with hydroxyl substitution in position 2, but allowing for extra substitutions in other places. We selected an array of salicylates bearing an additional individual substitution with a hydroxyl, methoxyl, methyl or chlorine group in all available positions, plus salicylates with an heteroatomic substitution with nitrogen in any position within the ring (pyridinic acids) (Additional file 1: Figure S1), and added them at 1 mM final concentration to the respective cultures, resulting in the production of several novel compounds, **8**–**19** (Figs. 4, 5) (Additional file 2: Figure S2, Tables S4–S11; Additional file 3: Figures S6–S13).

As expected, some of these compounds led to already characterized caboxamycins, such as the addition of SA behaving as a chemical complementation, restoring production of **1** and **2**; DHB yielding **3**; or 6MSA resulting in **6** (but, again, barely a hint of the putative acid form **7**). On the other hand, some reagents spawned no benzoxazoles in our culture conditions, such as 4-methoxysalicylate, which also seems to exert some hindering effect on the growth of this strain. Similarly, none of the pyridinic acids resulted in novel benzoxazoles, with the exception of 3-hydroxypicolinate (3HPA), a moiety shared by related benzoxazole A33853 [9], with the remarkable difference that here it appears as compounds **16** and **18**, sporting a complete benzoxazole feature, as opposed to the open, amide-linked structure of A33853. Furthermore, compounds **17** and **19**, which, like **5**, are PABA-derived, were unexpectedly obtained by the incorporation of 3HPA, a feat not readily observed with any of the other salicylates used in these assays. Moreover, it is unknown whether the methylation on the carboxylate in **19** could be a consequence of the same esterifying enzyme responsible for the biosynthesis of **2**.

Similarly to what occurs with compound **7**, compounds **11**, **12**, **13** and **15** (Fig. 5) represent peaks with distinctly benzoxazole-like absorption spectra (Additional file 2: Figure S2) and masses coherent with the expected products (*m/z* 270.08, 290.17, 290.15 and 304.08 [*M*+H]^+^, respectively), but these could not be adequately purified nor spectroscopically characterized due to low yields or presence of concomitant impurities, therefore remaining as putative novel caboxamycin derivatives and candidates for future experiments.

### Biological activity

All novel caboxamycin derivatives characterized in this work were tested against a selection of microorganisms (Gram-negative *Escherichia coli*, Gram-positive *Staphylococcus aureus*, *Micrococcus luteus* and *S. albus*, and yeast *Candida albicans*) via disk diffusion assay (Additional file 4: Table S12). Compounds **4**, **8** and **9** were found to inhibit growth in all Gram-positive bacteria, with **9** showing an additional mild inhibition of *C. albicans* growth. On the other hand, compound **14** was shown to selectively inhibit the growth of *S. aureus* at 5 µg.

These compounds were likewise submitted to cytotoxicity assays against tumor cell lines A549 (lung), MDA-MB-231 (breast), HT29 (colon), AGS (gastric) and A2780 (ovarian), using the murine fibroblast NIH/3T3 cell line as control (Additional file 4: Table S13). Most of the novel compounds show no activity under the selected cutoff level of 10 μM. However, compound **9** shows activity against all lines, which unfortunately includes the control cell line. On the other hand, compounds **4** and **8** could be of potential interest, as they seem to exert a selective cytotoxic effect against the gastric cell line AGS and ovarian cell line A2780 (compound **4**), as well as a mild action against the lung cell line A549.

## Discussion

The biosynthetic pathways of benzoxazole compounds nataxazole and caboxamycin show certain degree of flexibility since natural events of cross-talk with other pathways lead to hybrid compounds. In the case of nataxazole producer *Streptomyces* sp. Tü6176, the inactivation of polyketide synthase coding *natPK* led to the biosynthesis of benzoxazole UK-1 where the 6-methylsalicylic acid moiety had been substituted by salicylic acid, produced by the coelibactin biosynthesis pathway. Production of UK-1 can be improved growing the mutant strain in a zinc-deficient medium [8]. On the other hand, inactivation of *cbxA*, encoding a salicylate synthase, in caboxamycin producer *Streptomyces* sp. NTK937 led to the biosynthesis of 3′-hydroxycaboxamycin where the salicylate moiety had been substituted by 2,3-dihydroxybenzoate, produced by the enterobactin biosynthesis pathway [13]. The biosynthesis of these compounds represents a natural form of combinatorial production that prompted us to attempt the generation of novel benzoxazoles using combinatorial biosynthesis, heterologus expression and mutasynthesis, approaches that have been successful in many other cases [18–21].

**Fig. 6:**
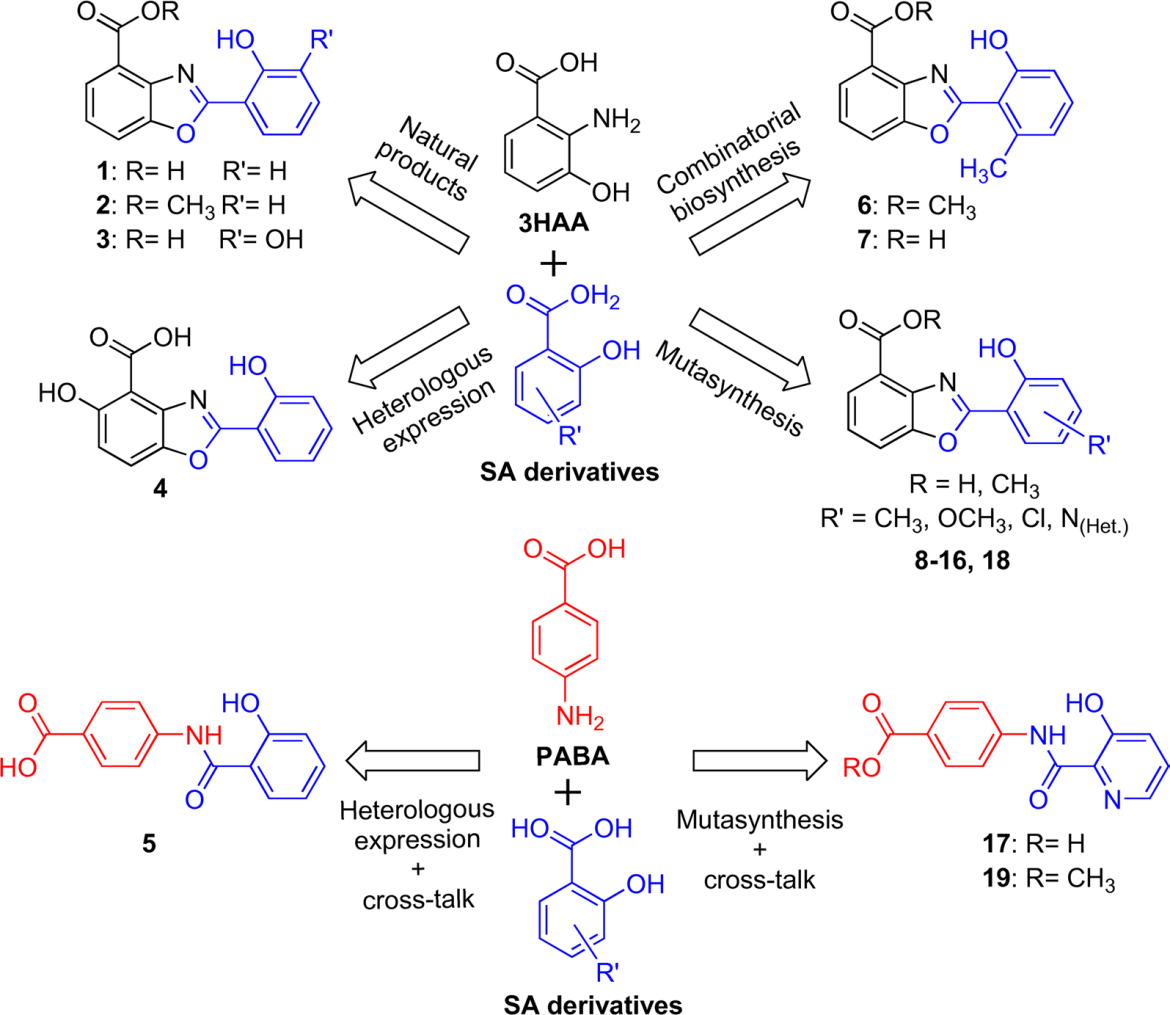
Schematic representation of the biosynthetic origin of compounds **1**–**19**

We have generated eleven novel caboxamycin derivatives (compounds **4**–**6**, **8**–**10**, **12**, **14** and **16**–**19**), and five putative novel derivatives (compounds **7**, **11**–**13** and **15**), (Fig. 6). The usual yield for wild-type cultures under same conditions is 1–2.5 mg for caboxamycin (**1**) and 0.2–0.7 mg for *O*-methylcaboxamycin (**2**). Compounds **4** and **5** (14.3 and 6.8 mg, respectively), generated by heterologous expression in *S. albus* J1074, were obtained in greater amounts due to the ermE*p-enforced expression of the corresponding biosynthetic genes, and the lack of regulatory controls from its original strain. Compound **6**, obtained by combinatorial biosynthesis in mutant strain ΔcbxA (0.2 mg), was produced at levels coherent with those of wild-type strain *Streptomyces* sp. NTK 937 for *O*-methylcaboxamycin (**2**). In the case of compounds **8**–**10**, **14** and **16**–**19**, these were isolated with varying success but at levels similar to those of *Streptomyces* sp. NTK 937, with higher quantities for the acid form than for the corresponding methyl ester. We also sought to improve mutasynthesis yields by ectopic expression of regulatory gene *cbxR* in the strain ΔcbxA/ΔentC. However, production levels did not increase accordingly as in prior uses of *cbxR* [13], possibly due to the lack of *cbxA*, which might contain the effecting region for CbxR, given the structural organization of the biosynthetic cluster.

Some of these novel caboxamycin derivatives show improved antibiotic and cytotoxic activities (compounds **4**, **8** and **9**). Especially remarkable is the fact that **4** can actually inhibit the growth of *S. albus*, the host strain where the compound was produced by combinatorial biosynthesis, a feat similar to that observed in the heterologous expression of the nataxazole cluster, whose unmethylated product, AJI9561, showed deleterious effects on the growth of the host strain [7]. On the other hand, the production of compound **4** in *S. albus* clearly shows the existence of orthologs for *cbxFGHI* within primary metabolism genes in *S. albus* strains, such as the biosynthetic pathway to aromatic amino acids [22, 23], secondary metabolism pathways, such as the biosynthesis of catecholate siderophores [24], or even alternative routes leading to 3HAA via NAD^+^ biosynthesis pathway from tryptophan catabolism [25]. In addition, hydroxylation of the 3HAA moiety of **4** might be mediated by *plm14* gene product, the immediate neighbor of the replaced genes in *S. albus* strain B29, which encodes a 3-hydroxybenzoate 6-hydroxylase activity that participates in paulomycins biosynthesis [16]. Otherwise, generation of **5** is serendipitous. It can only be surmised that stimulation of the chorismate pathway due to enforced salicylate biosynthesis by *cbxA* overexpression may activate other chorismate-derived routes such as PABA biosynthesis (Fig. 6), which naturally takes part as the first step towards folate biosynthesis [26]. Furthermore, PABA is a direct precursor of candicidins, polyene polyketides produced by *S. albus* J1074 [15]. No gene can be held responsible for the amide bond formation, since, to the best of our knowledge, no similar PABA-derived compounds of biological origin have been described in the literature, and non-ribosomal amide links can be achieved in several different ways [27]. On the other hand, the amide bond of compound **5** (Fig. 6) could be generated by CbxB that exerts a similar role during caboxamycin biosynthesis [13].

The production of some caboxamycin derivatives has also allowed confirming the roles previously assigned to some nataxazole biosynthesis genes [7]. That is the case for the generation of compound **6** by heterologous expression of polyketide synthase coding *natPK* in *Streptomyces* sp. NTK937, and the subsequent improvement of its yield by expressing AMP-dependant synthetase-ligase coding *natL1*, while the coexpression of *natPK* and *natL2* favored instead the production of **3**. These results are in consonance with the assigned roles of NatL1 and NatL2 in nataxazole biosynthesis: NatL2 was proposed to participate in the incorporation of the second 3HAA unit, while NatL1 might work on the condensation of 6MSA with 3HAA [7]. The small yields of **6** in ΔcbxA/pnatPK and ΔcbxA/pnatL2-PK might be due by the activity of CbxC.

Finally, mutasynthesis experiments revealed some unexpected results. Curiously, the addition of 2,5-dihydroxybenzoate leads to the production of **1** and **2**, through an unknown dehydroxylating mechanism that presumably acts after condensation of 2,5-dihydroxybenzoate to 3HAA, provided that no accumulation of SA occurs. Likewise, 3-methoxysalicylate gets spontaneously demethylated to DHB to become **3**, although in this instance, demethylation happens directly over the precursor, and DHB is indeed accumulated.

## Conclusions

The diversity of benzoxazoles generated by mutasynthesis, together with those obtained by heterologous expression of caboxamycin biosynthesis genes *cbxABCDE* in *S. albus* strains, and combinatorial biosynthesis experiments, show that the production of a wide variety of benzoxazoles could be potentially achieved by the sole expression of *cbxBCDE* genes (or orthologs thereof), supplying an external source of salicylate-like compounds, or with the concomitant expression of other genes capable of synthesizing salicylates, such as *cbxA* or *natPK*. The biological activity of the novel compounds generated, in comparison with that of the parental compound, can be altered and occasionally improved as shown in this work.

## Methods

### Strains, culture conditions and plasmids

*Streptomyces* sp. NTK937 [2], producer of caboxamycin, source of caboxamycin cluster genes *cbxABCDE*; *Streptomyces* sp. NTK937 gene-deleted mutant strains ΔcbxA and ΔcbxA/ΔentC [13] were used respectively for heterologous expression of nataxazole cluster genes and for mutasynthesis; *Streptomyces* sp. Tü6176 [4], producer of nataxazole, source of *natPK*, *natL1* and *natL2* genes; *S. albus* J1074 [14] and *S. albus* B29 [15] were used as heterologous hosts for the expression of caboxamycin biosynthesis genes; *E. coli* DH10B (Invitrogen) and *E. coli* ET12567 (pUB307) [28] were used for subcloning and intergeneric conjugation, respectively.

*Escherichia coli* culture media LB and 2xTY were used as previously described [29]. Tryptone Soy Broth (TSB) was used for culture of *Streptomyces* spp., MA for conjugation and sporulation, and R5A for secondary metabolite production [30]. Mutasynthesis assays were carried out by adding a final concentration of 1 mM of each salicylate-like compound (from a 1 M stock, dissolved in DMSO) at the same time of inoculation with the bacterial pre-culture (Additional file 1: Figure S1).

Two plasmids were used: pSETeTc [7] for integrative expression of the *cbxABCDE* genes in *S. albus* strains, and pEM4T [31] for the expression of nataxazole cluster genes in ΔcbxA strain. pCR-Blunt (Invitrogen) was used for routine PCR product cloning for verification purposes.

Culture media were supplemented with their due antibiotic when plasmid-bearing strains were used: ampicillin (100 µg/mL), apramycin (100 µg/mL for *E. coli*, 25 µg/mL for *Streptomyces*), thiostrepton (50 µg/mL), kanamycin (25 µg/mL), tetracycline (10 µg/mL), chloramphenicol (25 µg/mL), and/or nalidixic acid (50 µg/mL).

### DNA manipulation

DNA manipulations were performed according to standard procedures for *E. coli* [29] and *Streptomyces* [28]. PCR amplifications were carried out with Herculase II Fusion DNA Polymerase (Agilent Technologies) following an optimized standard PCR procedure on a SureCycler 8800 thermocycler (Agilent Technologies): initial denaturation at 99.9 °C for 2 min, 30 cycles comprised of 99.9 °C denaturation for 10 s, 65 °C annealing for 20 s, and 72 °C elongation at 30 s per kb of DNA to be amplified, plus an extra final cycle of 72 °C for 3 min. Products of the expected size were cloned into pCR-Blunt for sequence verification. PCR products were subsequently cloned into appropriate vectors using the selected restriction sites incorporated in the oligonucleotides.

### Construction of plasmids

Plasmid pSET-cbxABCDE was built from a 6 kb PCR fragment containing caboxamycin genes *cbxABCDE*. The PCR product was generated by using BamHI-flanked SS/fw oligonucleotide (5′-AA**GGATCC**GATCTGCGACGTGTCGCCGTC-3′) and EcoRI-flanked oligonucleotide AH/rv (5′-A**GAATTC**GGTGTTGTCGGGCATGGTG-3′). This fragment was subsequently purified, sequence-checked, and digested with the appropriate restriction enzymes, to be ligated into a BamHI/EcoRI-digested and dephosphorylated pSETeTc.

Constructions pnatPK and pnatL2-PK, both derived of pEM4T, for the expression of *natPK* or *natL2*, *natX* and *natPK*, respectively, were previously reported [9]. Plasmid pnatL1-PK, for simultaneous expression of *natPK* and *natL1*, was generated by blunt ended ligation of *natL1* at the BamHI site of pnatPK. The *natL1* gene was obtained as a PCR fragment using oligonucleotides ASL21/fw (5′-A**GAATTC**GTTCGTCTTCGGTCGGGAATGCG-3′) and ASL21/rv (5′-AA**GGATCC**GCGACCATGTCCTGCTGATCGG-3′).

### Analysis of metabolites by UPLC and HPLC–MS

Cultures of selected strains or mutants were extracted with ethyl acetate containing 1% formic acid (to enhance the extraction of compounds containing ionizing groups) and analysed by reverse phase chromatography with an Acquity UPLC instrument fitted with a BEH C18 column (1.7 µm, 2.1 × 100 mm, Waters), using acetonitrile (AcN) and aqueous 0.1% trifluoroacetic acid (TFA) as eluents. The program uses an isocratic hold of 10% AcN for 1 min, followed by a linear gradient up to 100% AcN over 7 min, at a flow rate of 0.5 mL/min and a column temperature of 35 °C.

For HPLC–MS analysis, an Alliance chromatographic system coupled to a ZQ4000 mass spectrometer and a SunFire C18 column (3.5 µm, 2.1 × 150 mm, Waters) was used. Solvents were the same as above and elution was performed with an initial isocratic hold with 10% AcN during 4 min followed by a linear gradient of AcN (10–88%) over 30 min, all at 0.25 mL/min. MS analysis was done by positive mode electrospray ionization (ESI), with a capillary voltage of 3 kV and a cone voltage of 20 V. Spectral identification and characterization of peaks was performed in both cases by photodiode array detection at 330 nm, using Empower software (Waters) to extract bidimensional chromatograms at different wavelengths, depending on the spectral characteristics of the desired compound.

### Isolation and structural characterization of compounds

Liquid production cultures were generally incubated at 30 °C and 250 rpm for 7 days and then 1 mL samples from each of the flasks were extracted with an equal volume of acidified ethyl acetate. Solid production cultures were carried out using 25-well plates with 1.5 mL solid R5A medium each and inoculated with a sterile cotton swab, then incubated at 30 °C for 7 days and extracted with an equal volume of acidified ethyl acetate. Both types of samples were subsequently vacuum-dried and redissolved in 50:50 DMSO:MeOH before chromatographic analysis.

Products **4**–**6**, **8**–**10**, **14** and **16**–**19** were isolated from 5 × 400 mL (in 2 L flasks) cultures whose supernatants were first filtered, then concentrated on a C18 cartridge (10 g, Waters), and subsequently fractioned on a 0.1% TFA–MeOH gradient. Fractions were submitted to UPLC analysis, and those containing desired compounds were dried *in vacuo*, resuspended in 50:50 DMSO:MeOH, and processed on a preparative HPLC SunFire C18 column (10 µm, 10 × 250 mm, Waters) using experimentally determined isocratic mixtures of 0.05% TFA with either AcN or MeOH at 5 mL/min. The purity of the isolated peaks was determined by HPLC–MS before structural elucidation. The isolated compounds were then dried *in vacuo*, resuspended in 50:50 *tert*-butanol:water and lyophilised. Yield and productivity of each compound was as follows: **4**, 14.3 mg (7.15 µg/mL); **5**, 6.8 mg (4.2 µg/mL); **6**, 0.2 mg (0.1 µg/mL); **8**, 1.6 mg (0.8 µg/mL); **9**, 2.1 mg (1.05 µg/mL); **10**, 0.5 mg (0.25 µg/mL); **14**, 2.5 mg (1.25 µg/mL); **16**, 1.3 mg (0.65 µg/mL); **17**, 0.7 mg (0.35 µg/mL); **18**, 0.3 mg (0.15 µg/mL); and **19**, 0.5 mg (0.15 µg/mL).

Structural elucidation was carried out at Fundación Medina (Granada, Spain) by a combination of ^1^H, ^13^C, COSY and HSQC experiments using DMSO-*d*_*6*_ as solvent (Additional file 2: Figure S2, Tables S1–S11). LC-DAD-HRMS analysis was carried out on an Agilent 1200 Rapid Resolution HPLC system coupled to a Bruker maXis mass spectrometer. For the NMR analysis the samples were dissolved in DMSO-*d*_*6*_ and transferred to a 1.7 mm tube. Acquisitions were carried out on a Bruker AVANCE III 500 MHz spectrometer equipped with a 1.7 mm TCI Microcryoprobe. All spectra (^1^H, ^13^C, COSY, HSQC, HMBC) were registered at 24 °C (Additional file 3: Figures S3–S13).

### Bioactivity assays

The antibiotic activities of **4**–**6**, **8**–**10**, **14** and **16**–**19** were analyzed with an antibiotic disk diffusion assay against *S. albus* J1074, *E. coli*, *S. aureus* and *M. luteus*. The antifungal activity was tested against *C. albicans*. In all cases 1, 2.5, 5, 10, and 20 µg of each compound were used and loaded on 6 mm paper disks. Plates were incubated overnight at 37 °C for *E. coli*, *S. aureus* and *M.luteus*, and at 30 °C for *S. albus* J1074 and *C. albicans* (Additional file 4: Table S12).

Cytotoxic activity of compounds **4**–**14** was tested against the following human tumor cell lines: colon adenocarcinoma (HT29), non-small cell lung cancer (A549), breast adenocarcinoma (MDA-MB-231), gastric carcinoma (AGS), and ovarian carcinoma (A2780). Mouse embryonic fibroblast cell line NIH/3T3 was used as control to evaluate cytotoxicity against non-malignant cells (Additional file 4: Table S13). Cells were previously grown for a week on DMEM-10%FBS medium, then aliquoted to 5000 cells per well in 96-well plates using the *Cell counting kit*-*8*-*(96992)* (Sigma-Aldrich) and grown for an extra 24 h. Compounds were dissolved in DMSO, keeping in mind that final concentration of DMSO in the assays should be kept at 0.1%. After the incubation, 10 μL of compound (in diverse concentrations) were added to each well and incubated for another 48 h. Lastly, 10 μL of CCK-8 reagent (Sigma-Aldrich) were added, left to develop for 2 h in the incubator, and measured at 450 nm using an *Elisa Bio*-*tek ELx 800* (BioTek).

## Additional files

**Additional file 1.** Compounds used for mutasynthesis experiments.

**Additional file 2.** Spectroscopic data of compounds characterized in this work.

**Additional file 3.** NMR spectra of compounds characterized in this work.

**Additional file 4.** Biological assays results.
