# Supplementary figures and images for: Exploring the biocombinatorial potential of benzoxazoles: generation of novel caboxamycin derivatives
Source: Microb Cell Fact. 2017 May 25;16:93. doi: 10.1186/s12934-017-0709-6 (PMC5445379; doi:10.1186/s12934-017-0709-6)

**Figure S1. Compounds used for mutasynthesis experiments. “Sal.” = salicylate**

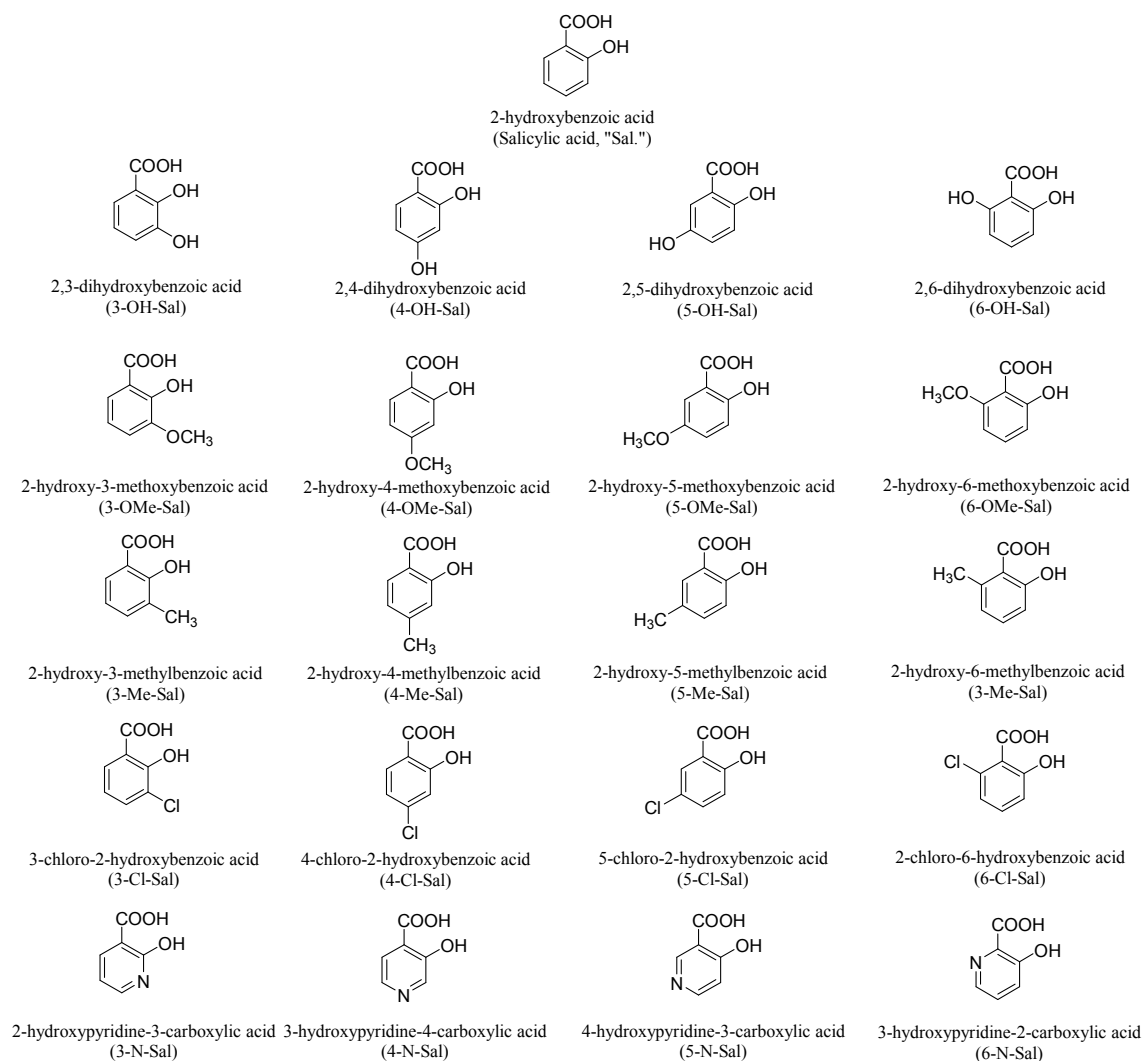

Supplement: Supplementary file 1 — Additional file 1. Compounds used for mutasynthesis experiments. [file 12934_2017_709_MOESM1_ESM.pdf]
