# Supplementary material for: Exploring the biocombinatorial potential of benzoxazoles: generation of novel caboxamycin derivatives
Source: Microb Cell Fact. 2017 May 25;16:93. doi: 10.1186/s12934-017-0709-6 (PMC5445379; doi:10.1186/s12934-017-0709-6)
Supplement: Supplementary file 2 — Additional file 2. Spectroscopic data of compounds characterized in this work. [file 12934_2017_709_MOESM2_ESM.pdf]

**Spectroscopic data of compounds characterized in this work**

**Figure S2. Absorption spectra of compounds**

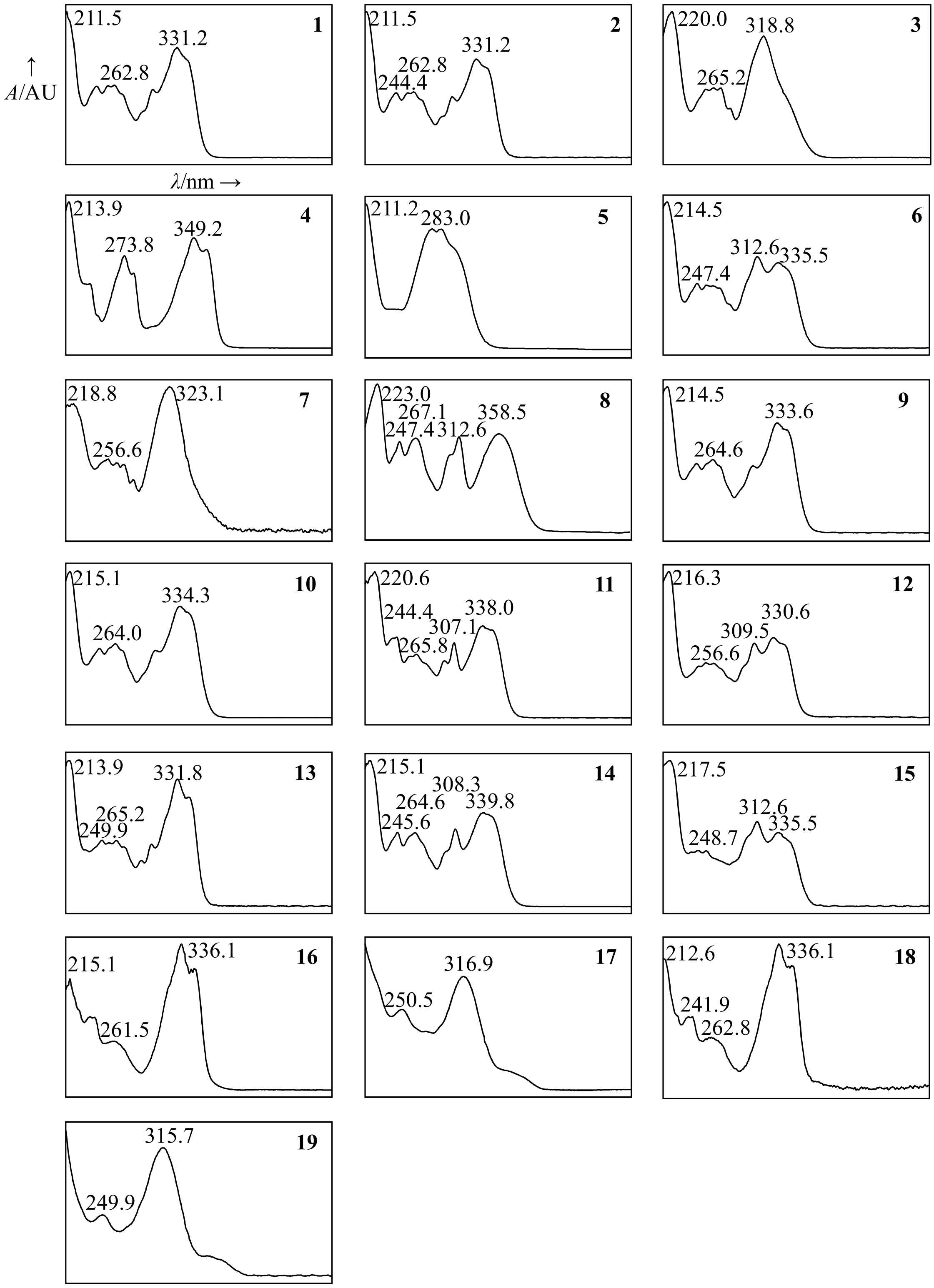

**Table S1. HRMS, $^{13}C$ and $^{1}H$ NMR data of 4 (DMSO-$d_6$, 500/125 MHz, 24ºC).**

Compound **4**: 5’-hydroxycaboxamycin
Formula: $C_{14}H_9NO_5$
$[M+H]^+$= 272.0558 (*calcd. 272.0553*)

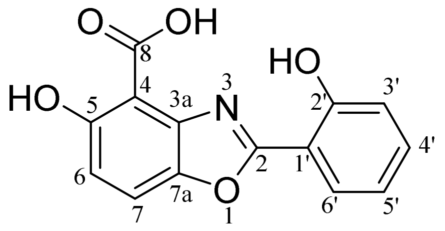


| Position | δ $^{13}C$ (ppm) | δ $^{1}H$ (ppm) | Mult. | $J$ (Hz) |
|---|---|---|---|---|
| 1’ | 109.7 | - | | |
| 2’ | 157.9 | - | | |
| 3’ | 117.0 | 7.12 | d | 8.2 |
| 4’ | 134.0 | 7.53 | ddd | 8.2, 7.6, 1.2 |
| 5’ | 121.7 | 7.08 | dd | 7.6, 7.6 |
| 6’ | 126.9 | 7.98 | m | |
| 2 | 163.5 | - | | |
| 3a | 138.6 | - | | |
| 4 | 103.7 | - | | |
| 5 | 159.6 | - | | |
| 6 | 125.6 | 7.04 | d | 8.9 |
| 7 | 116.2 | 7.98 | m | |
| 7a | 150.4 | - | | |
| 8 | 165.2 | - | | |
| OH (2’) | - | 12.00 | s | |
| OH (5) | - | n.d. | | |
| CO<u>OH</u> | - | 11.88 | br s | |
| $^{13}C$ δ indirectly determined from HSQC and HMBC experiments (n.d. = not determined) | | | | |

**Table S2. HRMS, $^{13}C$ and $^{1}H$ NMR data of 5 (DMSO-$d_6$, 500/125 MHz, 24ºC).**

Compound **5**: *para*-(salicilamido)benzoic acid

Formula: $C_{14}H_{11}NO_4$

$[M+H]^+$= 258.0763 (*calcd. 258.0761*)

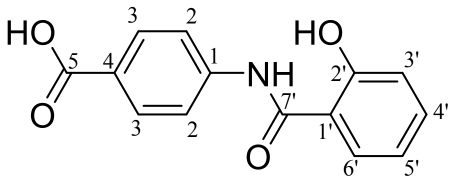


| Position | δ $^{13}C$ (ppm) | δ $^{1}H$ (ppm) | Mult. | $J$ (Hz) |
|---|---|---|---|---|
| 1’ | 117.9 | - | | |
| 2’ | 157.6 | - | | |
| 3’ | 116.9 | 7.00 | d | 8.2 |
| 4’ | 133.5 | 7.44 | ddd | 8.2, 7.6, 1.2 |
| 5’ | 119.0 | 6.97 | dd | 7.6, 7.6 |
| 6’ | 129.2 | 7.93 | dd | 7.7, 1.3 |
| 7’ | 166.2 | - | | |
| 1 | 142.2 | - | | |
| 2 | 119.6 | 7.85 | d | 8.6 |
| 3 | 130.1 | 7.95 | d | 8.6 |
| 4 | 125.6 | - | | |
| 5 | 166.7 | - | | |
| NH | - | 10.6 | br s | |
| OH | - | 11.9 | br s | |
| CO<u>OH</u> | - | 12.2 | br s | |
| $^{13}C$ δ indirectly determined from HSQC and HMBC experiments | | | | |

**Table S3. HRMS, $^{13}C$ and $^{1}H$ NMR data of 6 (DMSO-$d_6$, 500/125 MHz, 24ºC).**

Compound **6**: 6’,O-dimethylcaboxamycin

Formula: $C_{16}H_{13}NO_4$

$[M+H]^+$= 284.0914 (*calcd. 284.0917*)

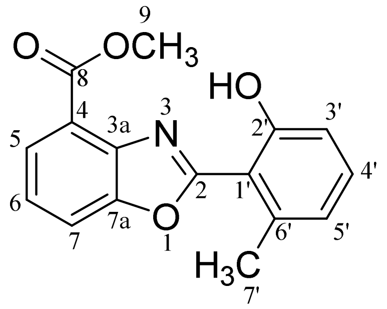


| Position | δ $^{13}C$ (ppm) | δ $^{1}H$ (ppm) | Mult. | *J* (Hz) |
|---|---|---|---|---|
| 1’ | 111.5 | - | | |
| 2’ | 159.4 | - | | |
| 3’ | 115.0 | 6.95 | d | 8.1 |
| 4’ | 120.3 | 7.39 | dd | 7.9, 7.9 |
| 5’ | 122.4 | 6.91 | d | 7.3 |
| 6’ | 139.6 | - | | |
| 7’ | 22.1 | 2.59 | br s | |
| 2 | n.d. | - | | |
| 3a | 138.7 | - | | |
| 4 | 121.0 | - | | |
| 5 | 127.2 | 8.02 | d | 7.8 |
| 6 | 125.6 | 7.60 | dd | 8.0, 8.0 |
| 7 | 116.2 | 8.15 | d | 8.0 |
| 7a | 150.4 | - | | |
| 8 | 165.2 | - | | |
| 9 | 52.7 | 3.96 | s | |
| OH | - | 11.92 | br s | |

$^{13}C$ δ indirectly determined from HSQC and HMBC experiments
(n.d. = not determined)

**Table S4. HRMS, $^{13}C$ and $^{1}H$ NMR data of 8 (DMSO-$d_6$, 500/125 MHz, 24ºC).**

Compound **8**: 5’-methoxycaboxamycin
Formula: $C_{15}H_{11}NO_5$
$[M+H]^+$= 286.0713 (*calcd. 286.0710*)

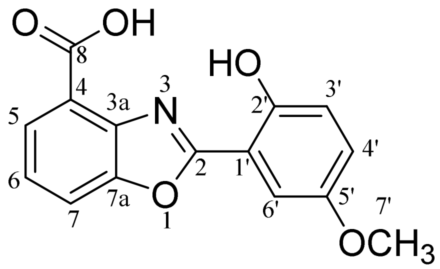


| Position | δ $^{13}C$ (ppm) | δ $^{1}H$ (ppm) | Mult. | *J* (Hz) |
|---|---|---|---|---|
| 1’ | 109.1 | - | | |
| 2’ | 152.6 | - | | |
| 3’ | 118.4 | 7.08 | br d | 8.9 |
| 4’ | 122.1 | 7.18 | br dd | 8.9, 1.7 |
| 5’ | 152.0 | - | | |
| 6’ | 109.4 | 7.48 | br d | 1.8 |
| 7’ | 55.5 | 3.82 | s | |
| 2 | 163.2 | - | | |
| 3a | 138.6 | - | | |
| 4 | 121.8 | - | | |
| 5 | 127.0 | 7.98 | d | 7.7 |
| 6 | 125.1 | 7.57 | dd | 7.7, 7.7 |
| 7 | 115.0 | 8.04 | d | 7.7 |
| 7a | 149.3 | - | | |
| 8 | 165.4 | - | | |
| OH (2’) | - | 11.4 | br s | |
| CO<u>OH</u> | - | 13.1 | br s | |
| $^{13}C$ δ indirectly determined from HSQC and HMBC experiments | | | | |

**Table S5. HRMS, $^{13}$C and $^{1}$H NMR data of 9 (DMSO-$d_6$, 500/125 MHz, 24ºC).**

Compound **9**: 4'-methylcaboxamycin
Formula: $C_{15}H_{11}NO_4$
$[M+H]^+$= 270.0766 (*calcd. 270.0761*)

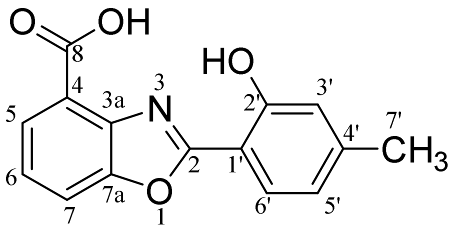


| Position | δ $^{13}$C (ppm) | δ $^{1}$H (ppm) | Mult. | $J$ (Hz) |
|---|---|---|---|---|
| 1' | 107.0 | - | | |
| 2' | 158.7 | - | | |
| 3' | 117.3 | 6.97 | m | |
| 4' | 145.1 | - | | |
| 5' | 120.9 | 6.92 | m | |
| 6' | 127.0 | 7.92 | m | |
| 7' | 21.2 | 2.36 | br s | |
| 2 | 163.3 | - | | |
| 3a | 138.6 | - | | |
| 4 | 122.3 | - | | |
| 5 | 126.8 | 7.97 | m | |
| 6 | 124.9 | 7.58 | m | |
| 7 | 114.6 | 8.06 | m | |
| 7a | 149.3 | - | | |
| 8 | 165.5 | - | | |
| OH (2') | - | 11.8 | br s | |
| COOH | - | 11.6 | br s | |
| $^{13}$C δ indirectly determined from HSQC and HMBC experiments | | | | |

**Table S6. HRMS, $^{13}C$ and $^{1}H$ NMR data of 10 (DMSO-$d_6$, 500/125 MHz, 24ºC).**

Compound **8**: 4',O-dimethylcaboxamycin
Formula: $C_{16}H_{13}NO_4$
$[M+H]^+$= 284.0920 (*calcd. 284.0917*)

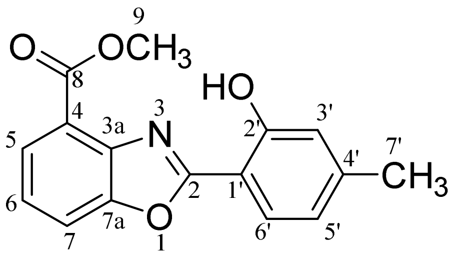


| Position | δ $^{13}C$ (ppm) | δ $^{1}H$ (ppm) | Mult. | *J* (Hz) |
|---|---|---|---|---|
| 1' | 106.9 | - | | |
| 2' | 158.2 | - | | |
| 3' | 117.3 | 6.99 | br s | |
| 4' | 145.3 | - | | |
| 5' | 121.0 | 6.94 | br d | 8.0 |
| 6' | 127.1 | 7.93 | d | 8.0 |
| 7' | 21.2 | 2.37 | br s | |
| 2 | 163.8 | - | | |
| 3a | 138.6 | - | | |
| 4 | 120.0 | - | | |
| 5 | 126.7 | 7.99 | br d | 7.7 |
| 6 | 125.0 | 7.58 | dd | 8.0 |
| 7 | 115.5 | 8.13 | br d | 8.0 |
| 7a | 149.3 | - | | |
| 8 | 164.4 | - | | |
| 9 | 52.0 | 3.96 | s | |
| OH (2') | - | 11.8 | br s | |
| $^{13}C$ δ indirectly determined from HSQC and HMBC experiments | | | | |

**Table S7. HRMS, $^{13}C$ and $^{1}H$ NMR data of 14 (DMSO-$d_6$, 500/125 MHz, 24ºC).**

Compound **14**: 5'-chlorocaboxamycin
Formula: $C_{14}H_8ClNO_4$
$[M+H]^+$= 290.0217 (*calcd. 290.0215*)

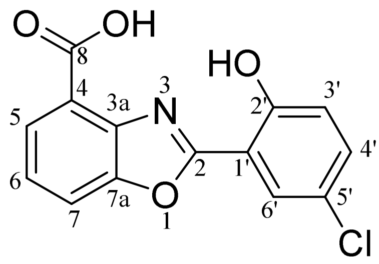


| Position | δ $^{13}C$ (ppm) | δ $^{1}H$ (ppm) | Mult. | *J* (Hz) |
|---|---|---|---|---|
| 1' | 111.9 | - | | |
| 2' | 157.5 | - | | |
| 3' | 119.9 | 7.19 | d | 8.5 |
| 4' | 134.4 | 7.59 | d | 8.2 |
| 5' | 123.8 | - | | |
| 6' | 127.1 | 8.01 | br s | |
| 2 | 162.8 | - | | |
| 3a | 139.0 | - | | |
| 4 | 122.8 | - | | |
| 5 | 127.8 | 8.00 | d | 7.9 |
| 6 | 126.2 | 7.60 | dd | 7.9, 7.9 |
| 7 | 115.8 | 8.11 | d | 7.8 |
| 7a | 150.2 | - | | |
| 8 | 166.0 | - | | |
| OH | - | 11.86 | br s | |
| CO<u>OH</u> | - | - | | |
| $^{13}C$ δ indirectly determined from HSQC and HMBC experiments | | | | |

**Table S8. HRMS, $^{13}C$ and $^{1}H$ NMR data of 16 (DMSO-$d_6$, 500/125 MHz, 24ºC).**

Compound **16**: 6’-azacaboxamycin
Formula: $C_{13}H_8N_2O_4$
$[M+H]^+$= 257.0560 (*calcd. 257.0557*)

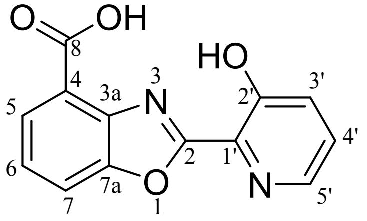


| Position | δ $^{13}C$ (ppm) | δ $^{1}H$ (ppm) | Mult. | *J* (Hz) |
|---|---|---|---|---|
| 1’ | 126.8 | - | | |
| 2’ | 163.4 | - | | |
| 3’ | 129.6 | 6.98 | dd | 8.8, 1.3 |
| 4’ | 129.5 | 7.30 | dd | 8.8, 4.0 |
| 5’ | 137.3 | 8.01 | dd | 4.0, 1.3 |
| 2 | 163.2 | - | | |
| 3a | 135.7 | - | | |
| 4 | 124.1 | - | | |
| 5 | 126.1 | 7.89 | d | 7.7 |
| 6 | 125.9 | 7.61 | dd | 7.9, 7.9 |
| 7 | 113.8 | 8.12 | d | 8.2 |
| 7a | 148.2 | - | | |
| 8 | 162.6 | - | | |
| OH | - | n.d. | | |
| CO<u>OH</u> | - | n.d. | | |

$^{13}C$ δ indirectly determined from HSQC and HMBC experiments
(n.d. = not determined)

**Table S9. HRMS, $^{13}$C and $^{1}$H NMR data of 17 (DMSO-$d_6$, 500/125 MHz, 24ºC).**

Compound **17**: *para*-(3-hydroxypicolinamido)benzoic acid
Formula: $C_{13}H_{10}N_2O_4$
$[M+H]^+$= 259.0714 (*calcd. 259.0713*)

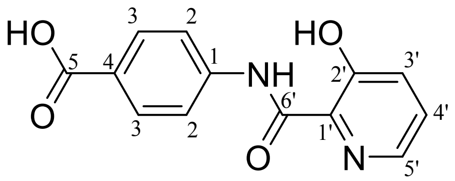


| Position | δ $^{13}$C (ppm) | δ $^{1}$H (ppm) | Mult. | *J* (Hz) |
|---|---|---|---|---|
| 1’ | 131.1 | - | | |
| 2’ | 157.3 | - | | |
| 3’ | 126.2 | 7.51 | dd | 8.5, 1.1 |
| 4’ | 129.5 | 7.62 | dd | 8.5, 4.3 |
| 5’ | 139.8 | 8.28 | br d | 3.0 |
| 6’ | n.d. | - | | |
| 1 | 141.0 | - | | |
| 2 | 119.4 | 8.00 | d | 8.6 |
| 3 | 129.9 | 7.95 | d | 8.6 |
| 4 | 126.3 | - | | |
| 5 | 166.7 | - | | |
| NH | - | 11.1 | br s | |
| OH | - | 12.7 | br s | |
| CO<u>OH</u> | - | 11.9 | br s | |
| $^{13}$C δ indirectly determined from HSQC and HMBC experiments (n.d. = not determined) | | | | |

**Table S10. HRMS, $^{13}C$ and $^{1}H$ NMR data of 18 (DMSO-$d_6$, 500/125 MHz, 24ºC).**

Compound **18**: 6'-aza-O-methylcaboxamycin
Formula: $C_{14}H_{10}N_2O_4$
$[M+H]^+$= 271.0712 (*calcd. 271.0713*)

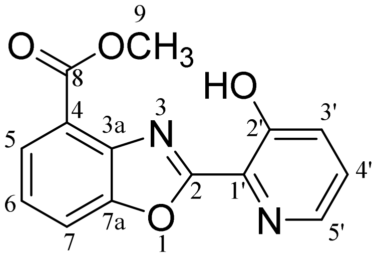


| Position | δ $^{13}C$ (ppm) | δ $^{1}H$ (ppm) | Mult. | *J* (Hz) |
|---|---|---|---|---|
| 1' | n.d. | - | | |
| 2' | n.d. | - | | |
| 3' | 125.2 | 7.62 | br s | |
| 4' | 128.2 | 7.60 | br s | |
| 5' | 141.4 | 8.36 | br s | |
| 2 | n.d. | - | | |
| 3a | 138.1 | - | | |
| 4 | 120.4 | - | | |
| 5 | 126.9 | 8.06 | d | 7.8 |
| 6 | 125.8 | 7.66 | dd | 7.8, 7.8 |
| 7 | 116.0 | 8.22 | d | 8.0 |
| 7a | 149.5 | - | | |
| 8 | 164.2 | - | | |
| 9 | 52.1 | 3.98 | s | |
| OH | - | 11.77 | br s | |

$^{13}C$ δ indirectly determined from HSQC and HMBC experiments
(n.d. = not determined)

**Table S11. HRMS, $^{13}$C and $^{1}$H NMR data of 19 (DMSO-$d_6$, 500/125 MHz, 24ºC).**

Compound **19**: methyl *para*-(3-hydroxypicolinamido)benzoate

Formula: $C_{14}H_{12}N_2O_4$

$[M+H]^+$= 273.0869 (*calcd. 273.0870*)

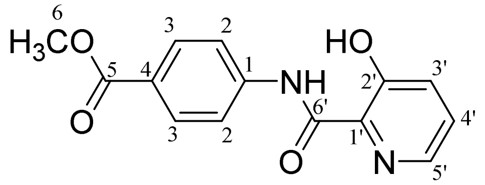


| Position | δ $^{13}$C (ppm) | δ $^{1}$H (ppm) | Mult. | *J* (Hz) |
|---|---|---|---|---|
| 1' | 131.0 | - | | |
| 2' | 157.3 | - | | |
| 3' | 126.1 | 7.51 | dd | 8.4, 1.1 |
| 4' | 129.4 | 7.62 | dd | 8.4, 4.2 |
| 5' | 139.7 | 8.29 | br d | 3.7 |
| 6' | n.d. | - | | |
| 1 | 141.4 | - | | |
| 2 | 120.4 | 8.03 | d | 8.6 |
| 3 | 129.7 | 7.98 | d | 8.7 |
| 4 | 125.0 | - | | |
| 5 | 165.4 | - | | |
| 6 | 51.7 | 3.96 | s | |
| NH | - | 11.13 | br s | |
| OH | - | 11.83 | br s | |

$^{13}$C δ indirectly determined from HSQC and HMBC experiments
(n.d. = not determined)
