# Supplementary material for: Exploring the biocombinatorial potential of benzoxazoles: generation of novel caboxamycin derivatives
Source: Microb Cell Fact. 2017 May 25;16:93. doi: 10.1186/s12934-017-0709-6 (PMC5445379; doi:10.1186/s12934-017-0709-6)

NMR spectra of compounds characterized in this work

Figure S3. ^{1}H-NMR and HSQC of 4 (DMSO-d_6, 500 MHz)

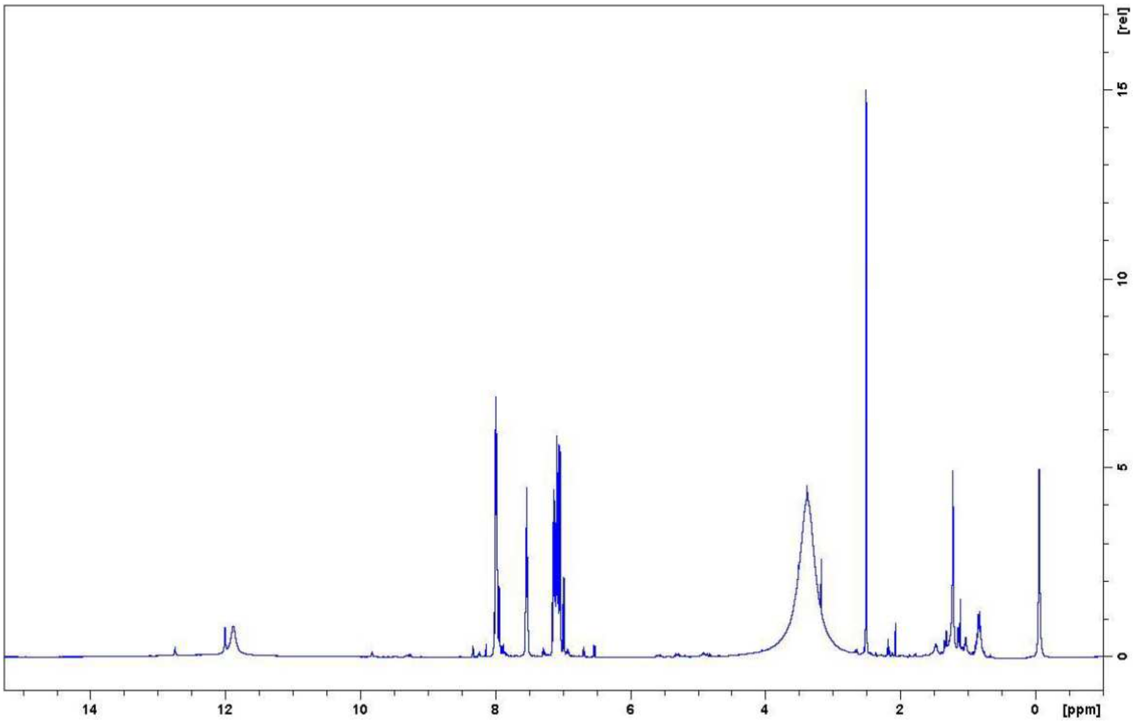

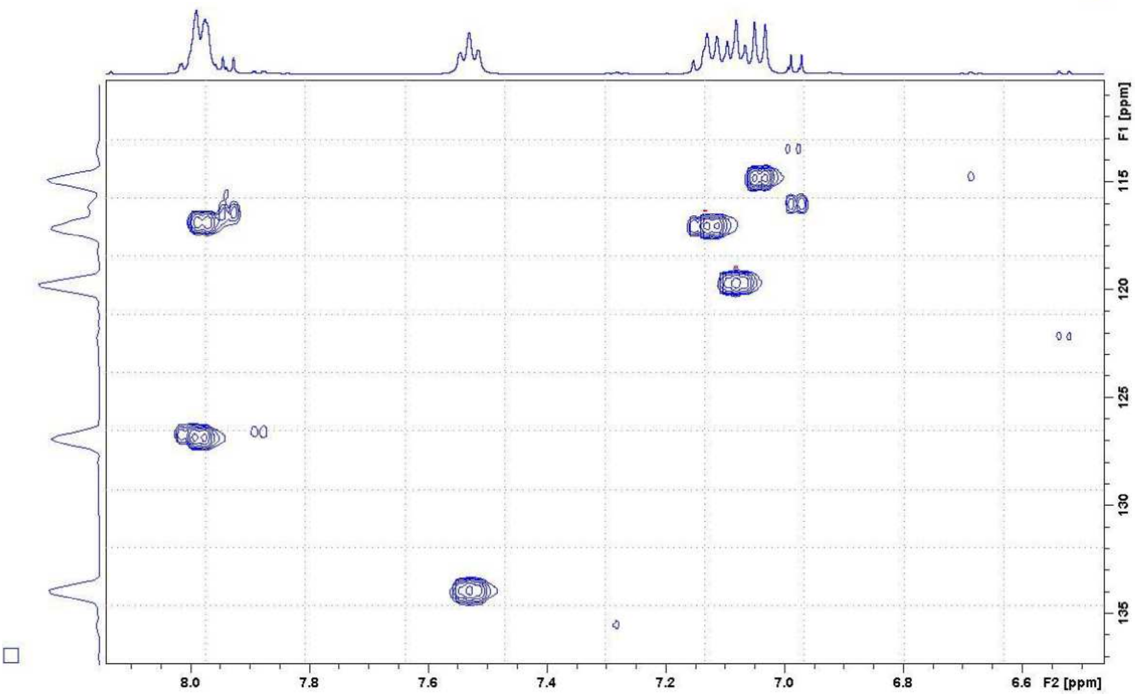

Figure S4. ^{1}H-NMR and HSQC spectra, and key HMBC correlations of 5 (DMSO-d_6, 500 MHz)

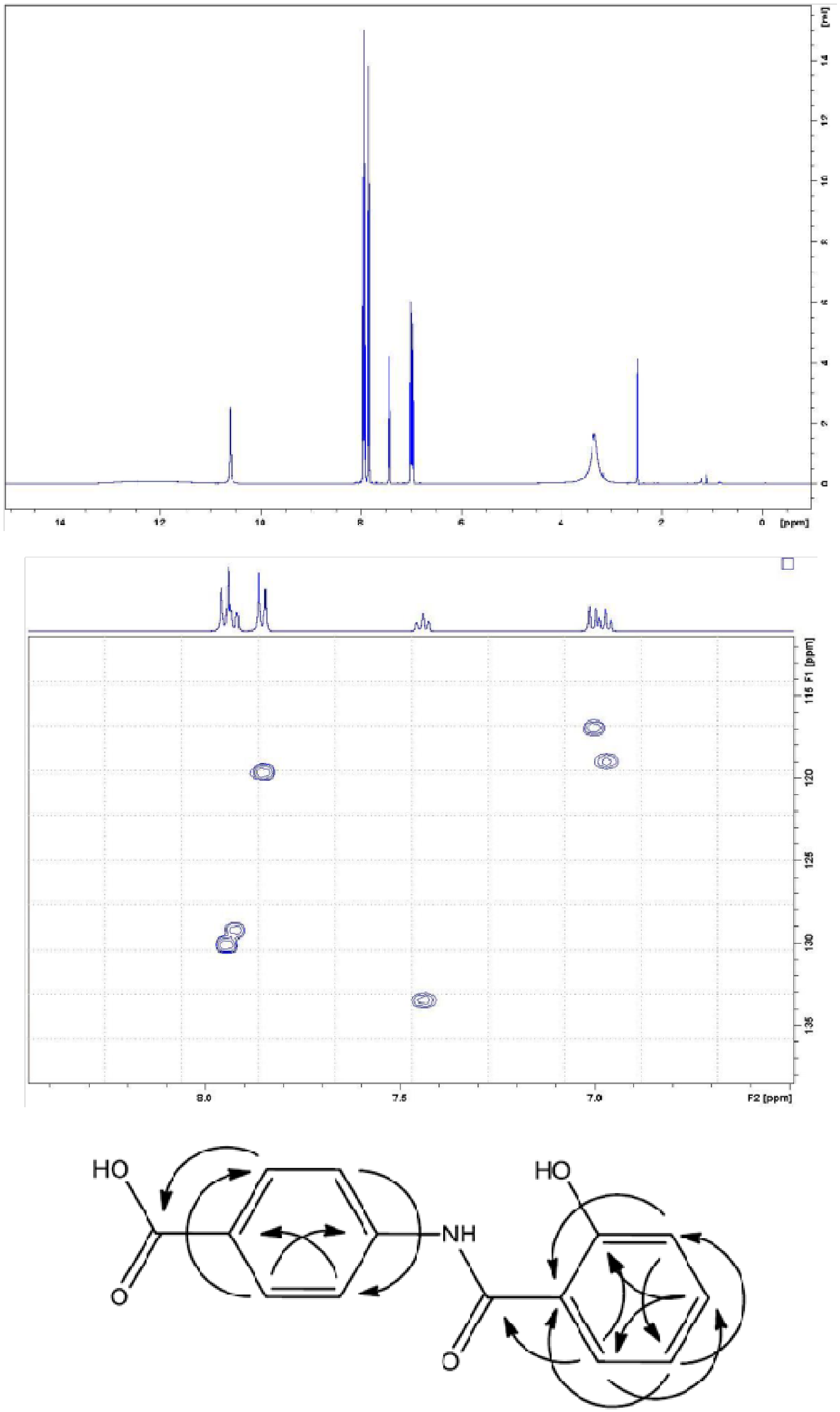

Figure S5. ^{1}H-NMR (with aromatic region expansion) and key NOESY and HMBC correlations of 6 (DMSO-d_6, 500 MHz)

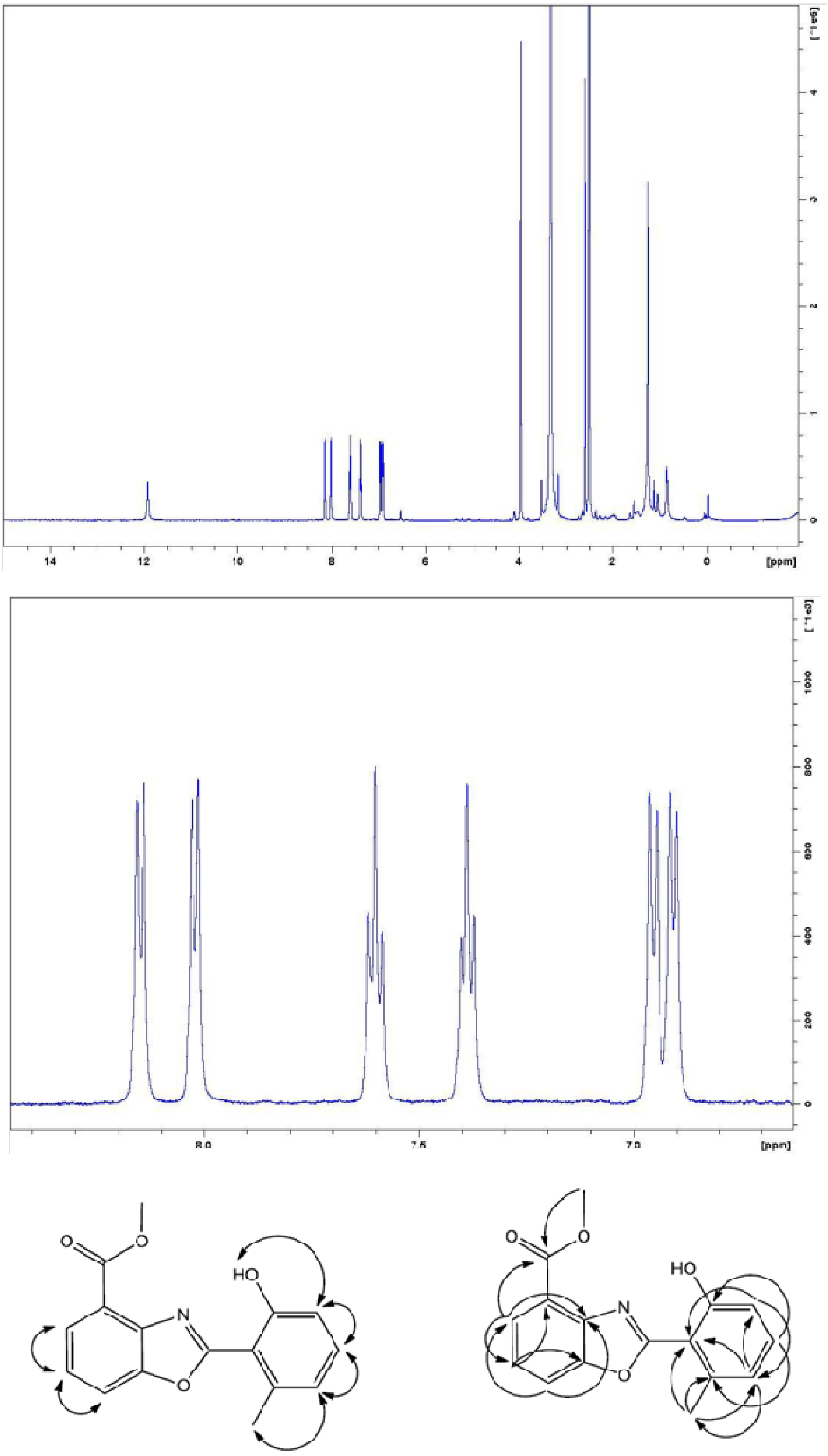

Figure S6. ^{1}H-NMR and HSQC spectra, and key HMBC correlations of 8 (DMSO-d_6, 500 MHz)

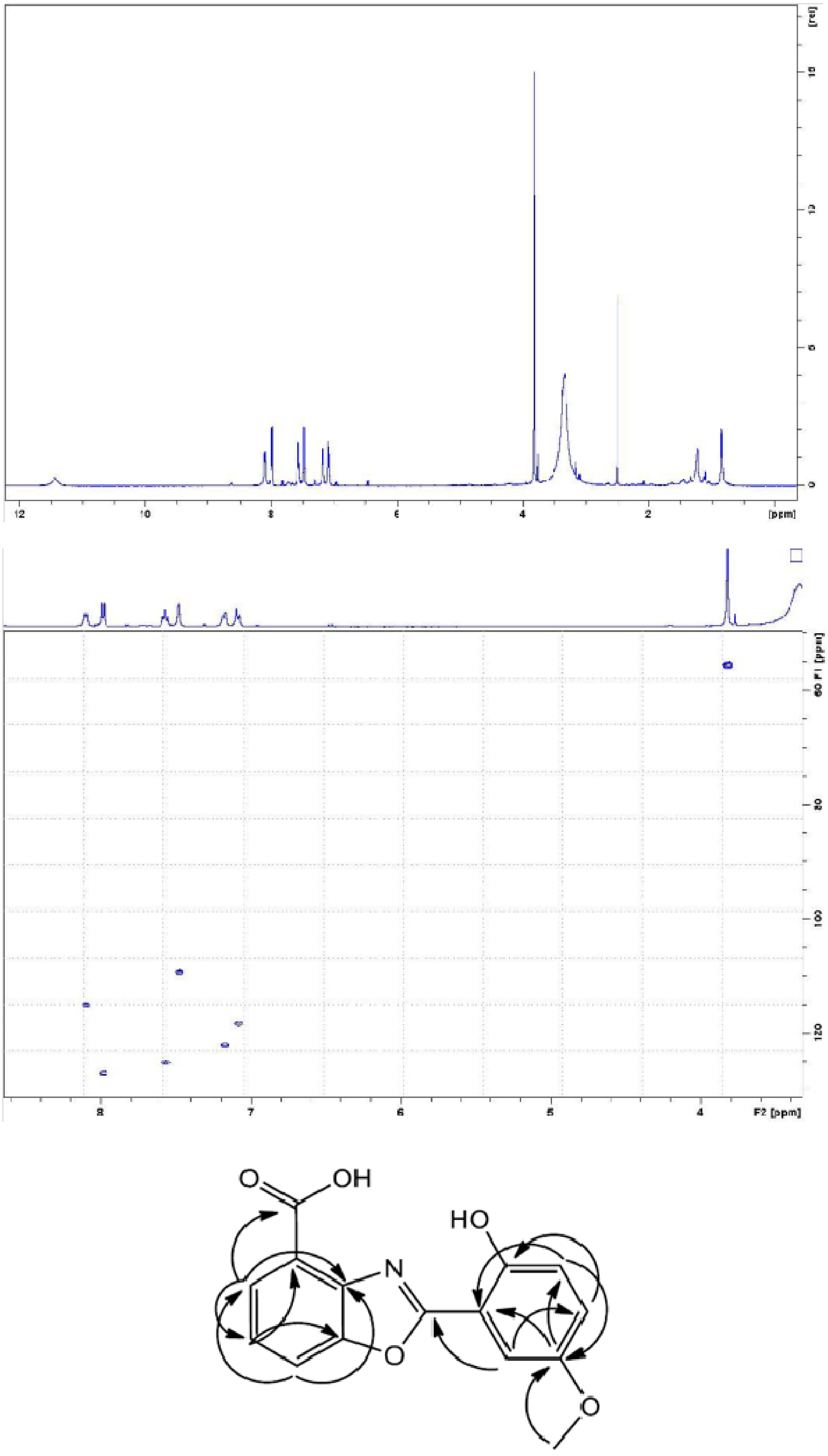

Figure S7. ^{1}H-NMR and HSQC spectra, and key HMBC correlations of 9 (DMSO-d_6, 500 MHz)

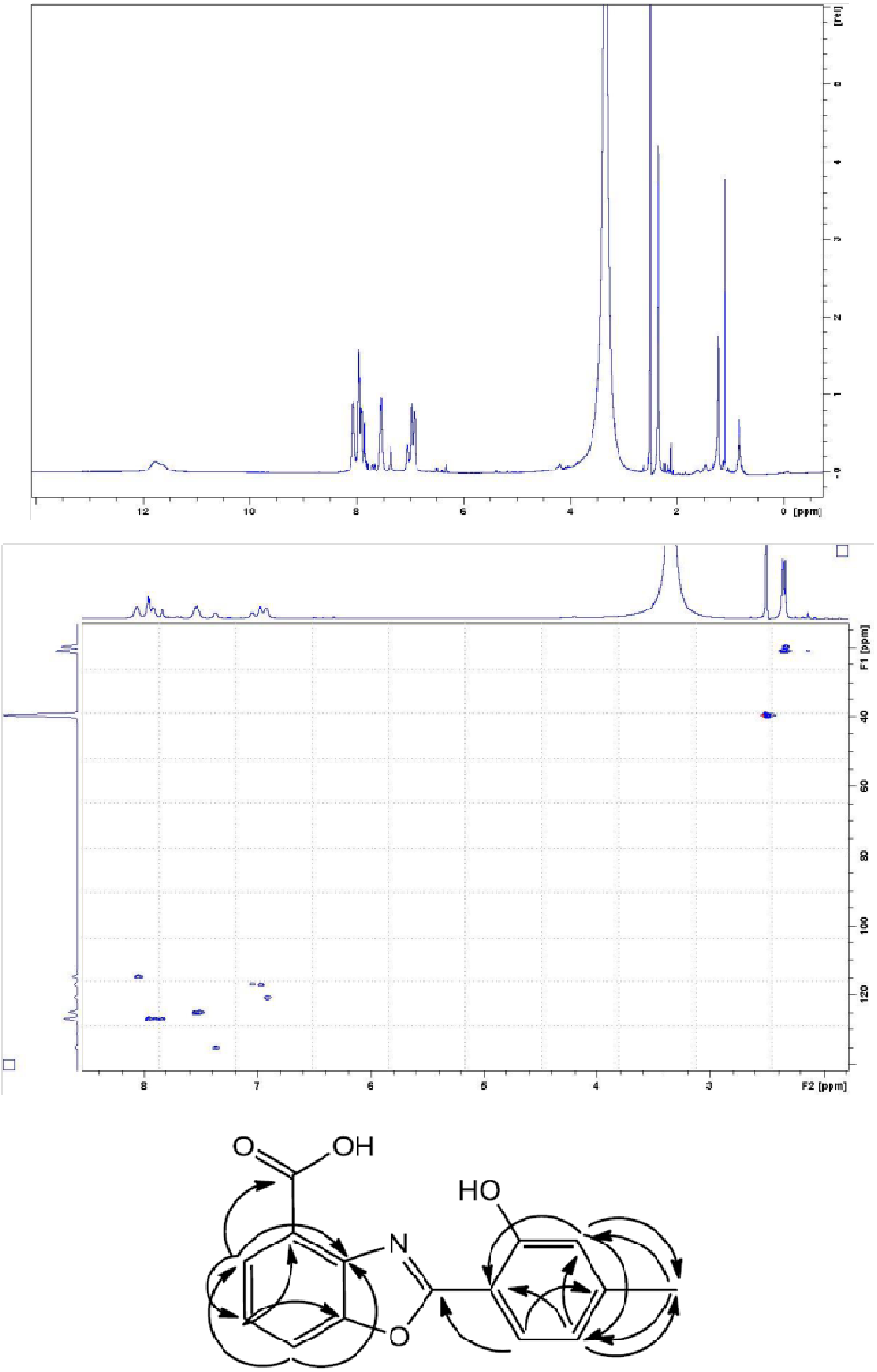

Figure S8. ^{1}H-NMR and HSQC spectra, and key HMBC correlations of 10 (DMSO-d_6, 500 MHz)

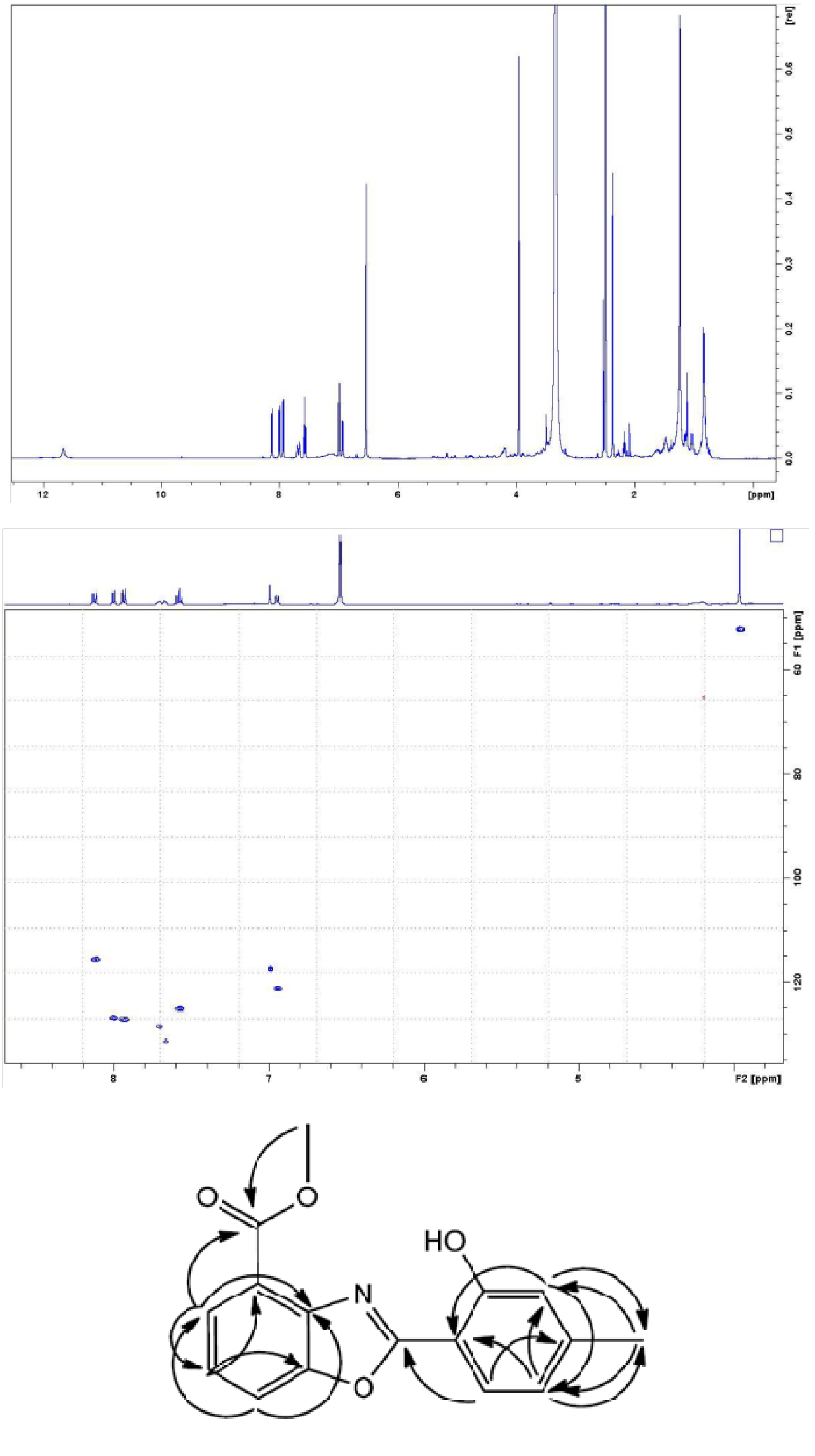

Figure S9. ^{1}H-NMR (with aromatic region expansion) and key HMBC correlations of 14 (DMSO-d_6, 500 MHz)

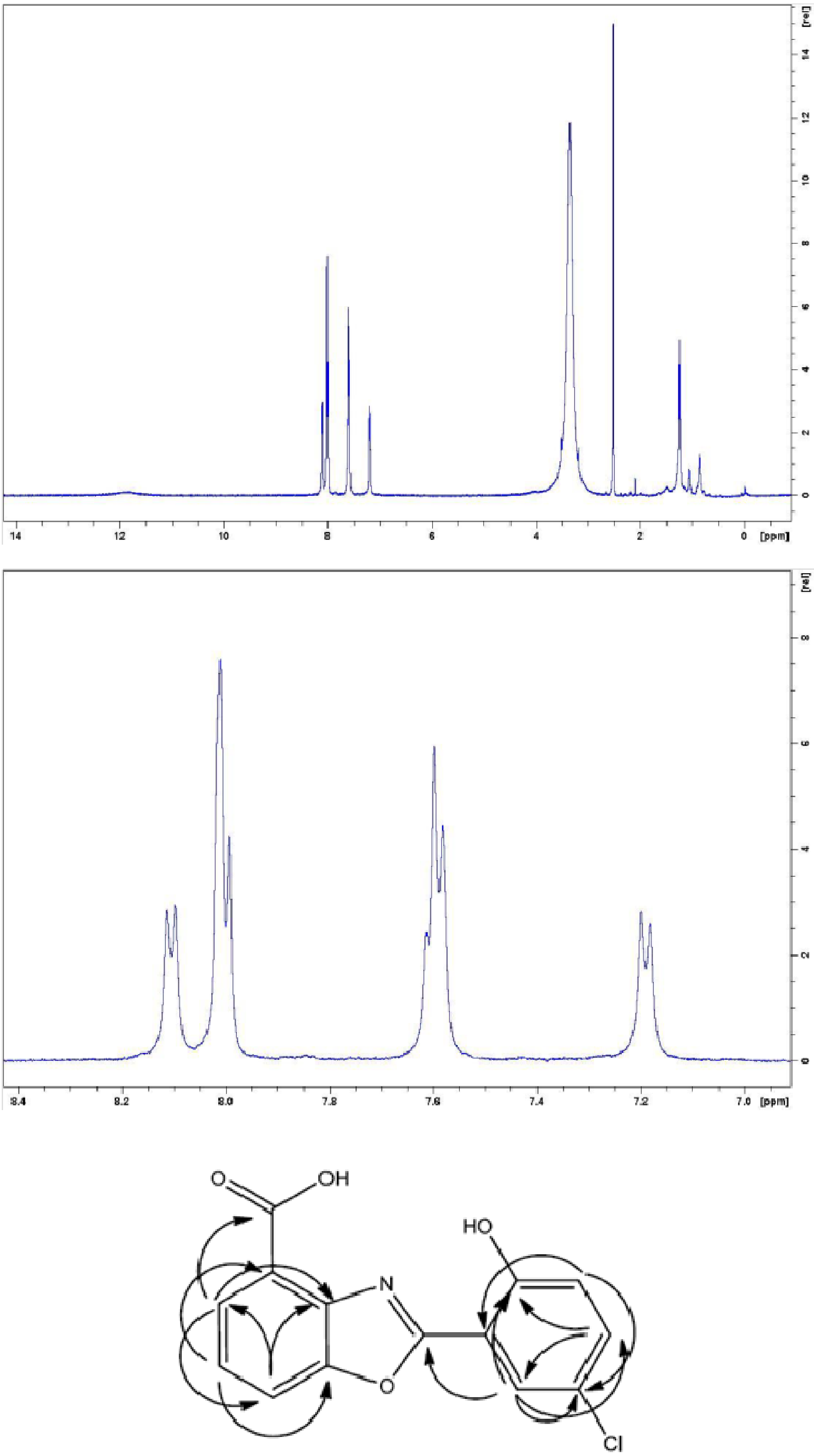

Figure S10. ^{1}H-NMR and HSQC spectra, and key HMBC correlations of 16 (DMSO-d_6, 500 MHz)

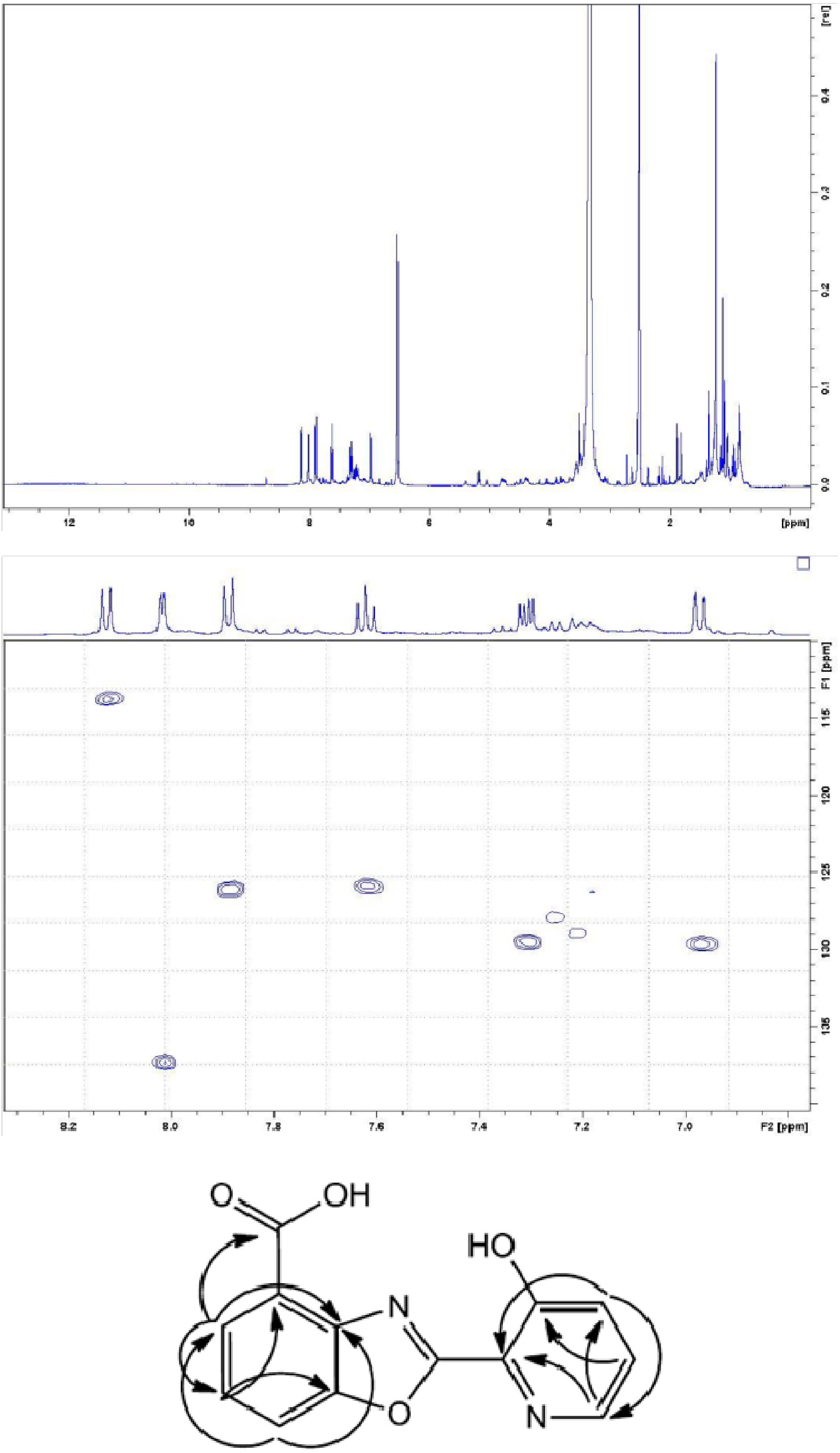

Figure S11. ^{1}H-NMR and HSQC spectra, and key HMBC correlations of 17 (DMSO-d_6, 500 MHz)

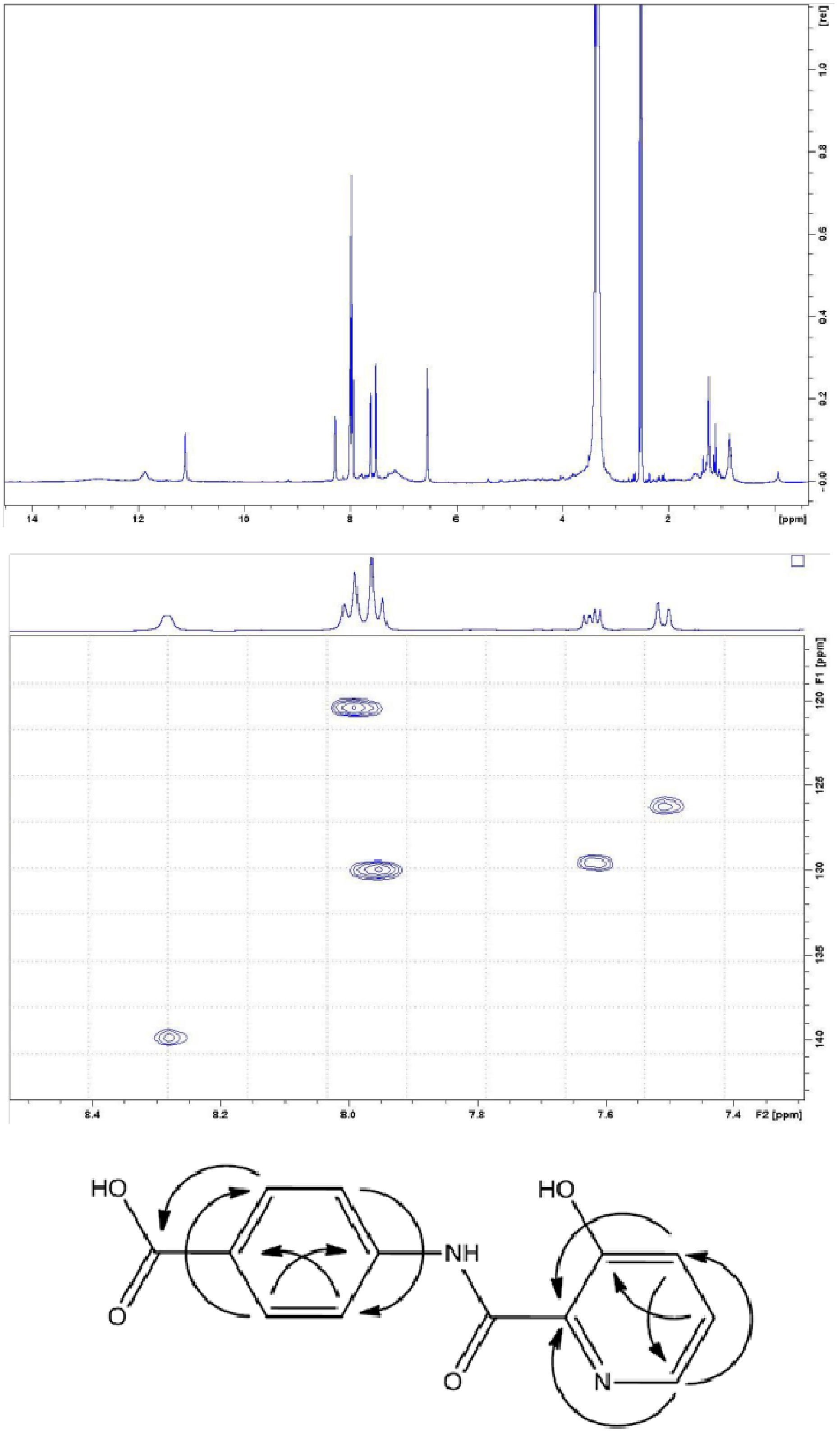

Figure S12.1. ^{1}H-NMR and HSQC spectra of 18 (DMSO-d_6, 500 MHz)

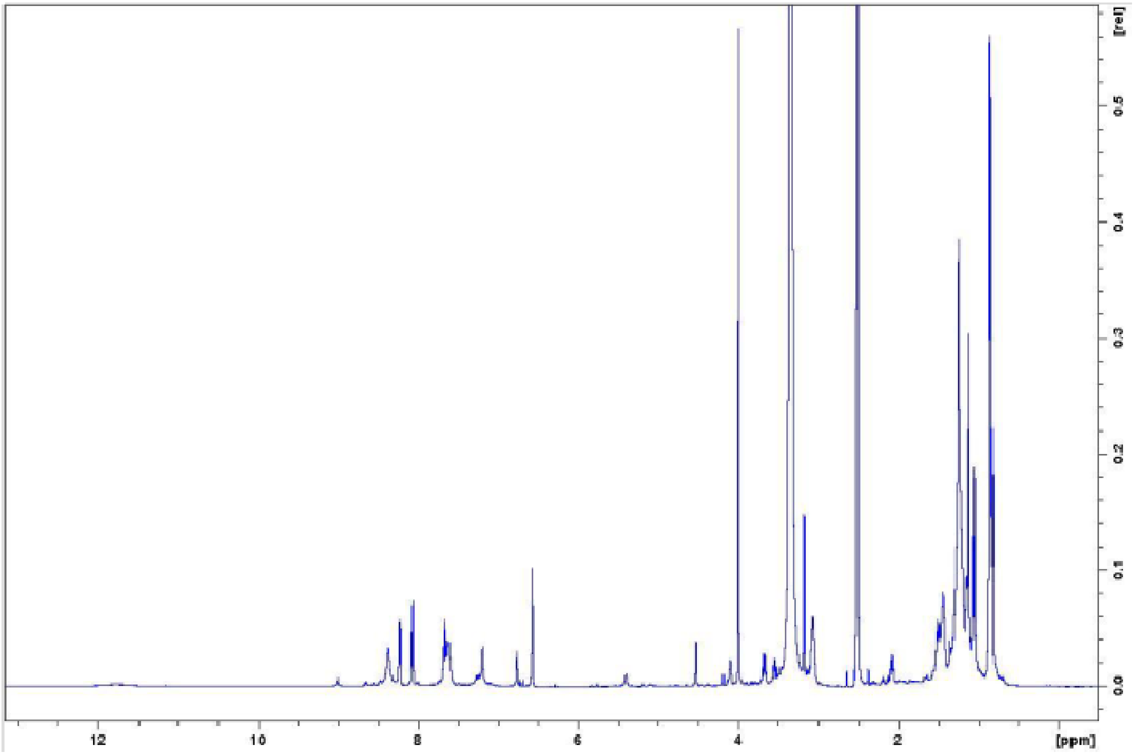

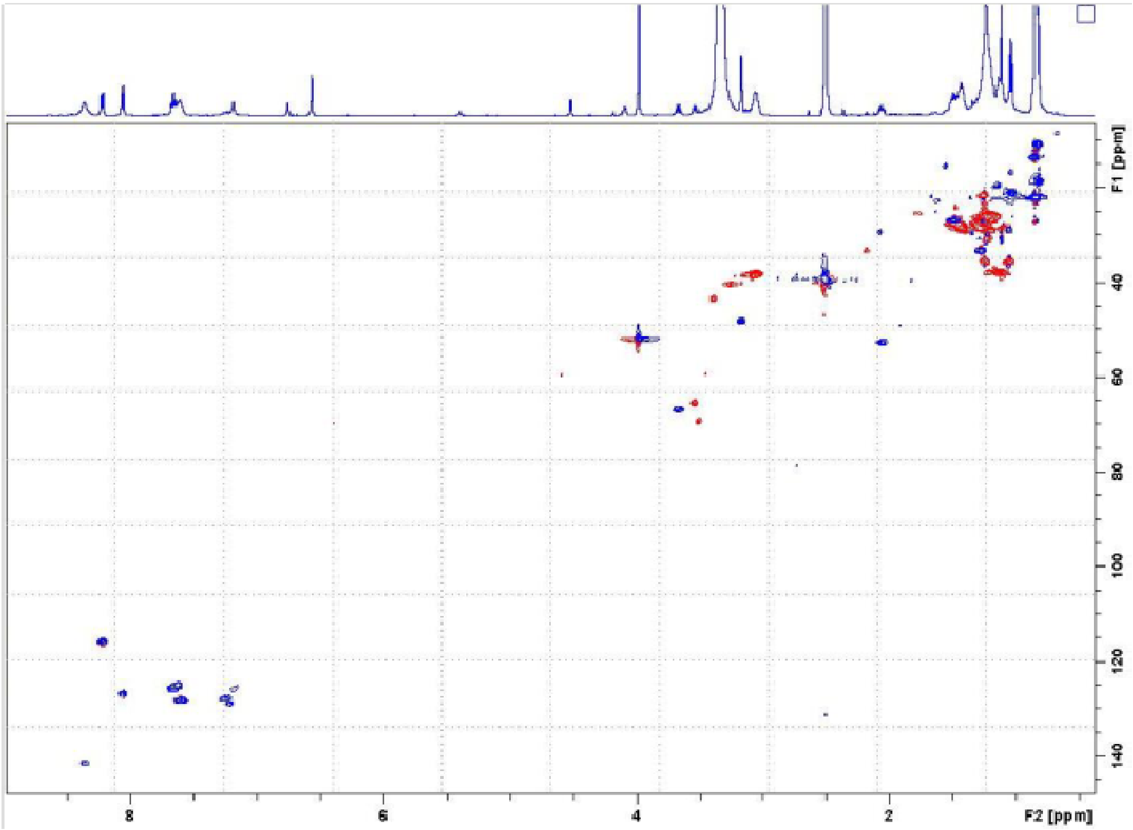

Figure S12.2. DOSY spectrum (with highlighted signals) of 18 (DMSO-d_6, 500 MHz)

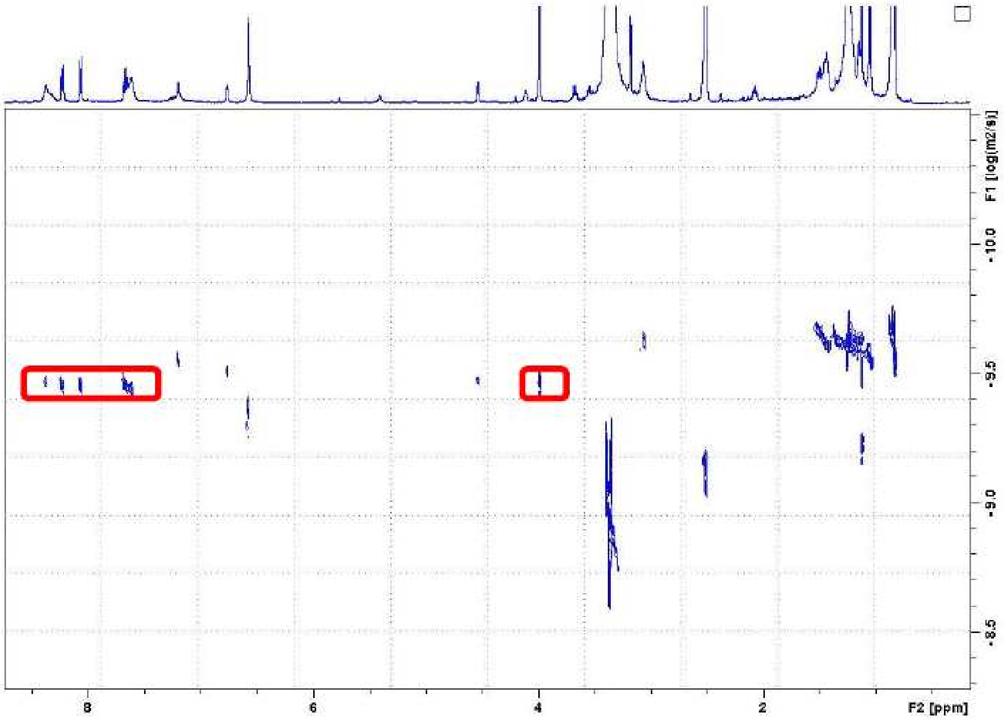

Figure S13. ^{1}H-NMR and HSQC spectra, and key HMBC correlations of 19 (DMSO-d_6, 500 MHz)

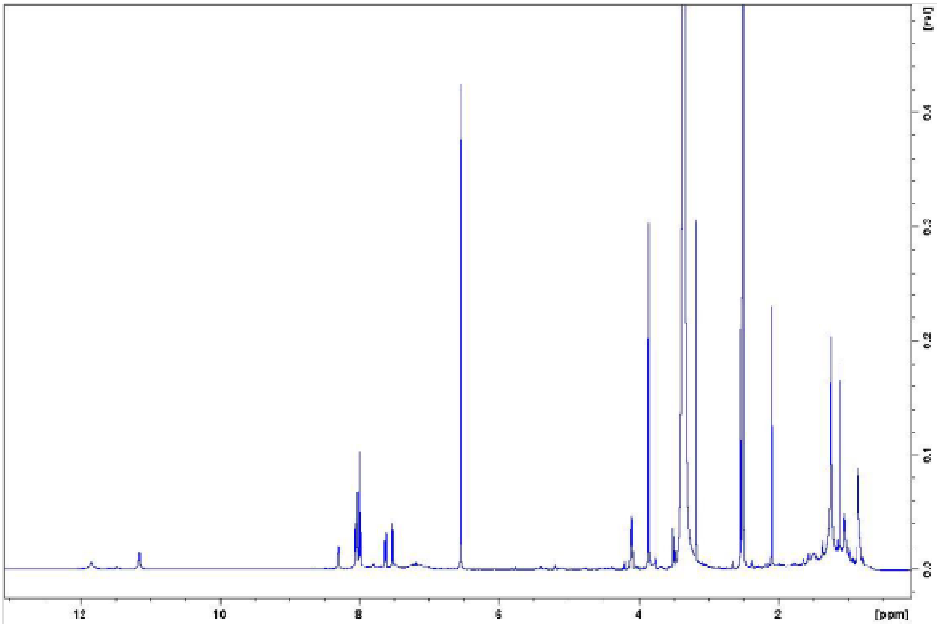

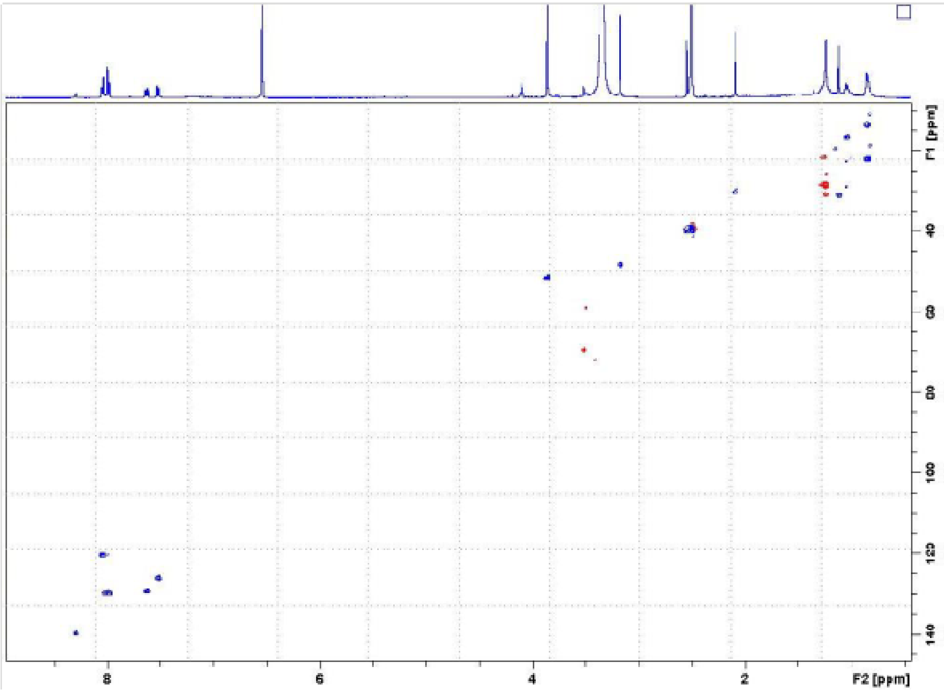

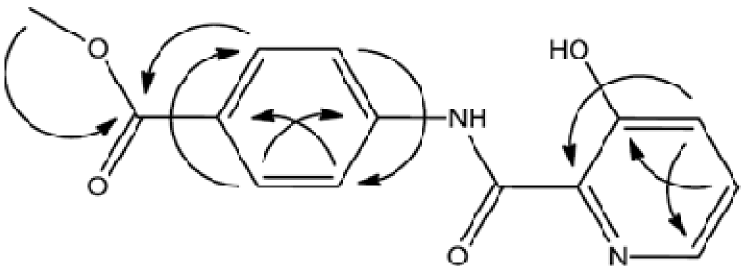

Supplement: Supplementary file 3 — Additional file 3. NMR spectra of compounds characterized in this work. [file 12934_2017_709_MOESM3_ESM.pdf]
