# Supplementary material for: Exploring the biocombinatorial potential of benzoxazoles: generation of novel caboxamycin derivatives
Source: Microb Cell Fact. 2017 May 25;16:93. doi: 10.1186/s12934-017-0709-6 (PMC5445379; doi:10.1186/s12934-017-0709-6)
Supplement: Supplementary file 4 — Additional file 4. Biological assays results. [file 12934_2017_709_MOESM4_ESM.pdf]

Biological assays results.

Table S12. Detailed results of disk diffusion antibiotic assays

Compound	Amount (µg)	Halo diameter (mm)	Against
**1**	2.5	9	*C. albicans*
**2**		Inactive	
**3**	10	9	*C. albicans*
**4**	10<br>5<br>5	9<br>8<br>8	*M. luteus*<br>*S. albus*<br>*S. aureus*
**5**		Inactive	
**6**		Inactive	
**8**	20<br>20<br>20	8<br>9<br>8	*M. luteus*<br>*S. albus*<br>*S. aureus*
**9**	10<br>10<br>5<br>10	8<br>10<br>9<br>9	*C. albicans*<br>*M. luteus*<br>*S. albus*<br>*S. aureus*
**10**		Inactive	
**14**	5	12	*S. aureus*
**16**		Inactive	
**17**		Inactive	
**18**		Inactive	
**19**		Inactive	

Table S13. Detailed results of cytotoxicity assays

Compound	Lung A549	Breast MDA-MB-231	Colon HT29	Gastric AGS	Ovarian A2780	Fibroblasts NIH/3T3
**1**	>10	>10	>10	>10	7.29	11
**2**	2.70	6.10	3.70	5.00	4.93	5.14
**3**	+	>10	+	+	>10	+
**4**	8.05	>10	>10	5.56	3.35	>10
**5**	>10	>10	>10	>10	>10	>10
**6**	+	>10	+	+	+	+
**8**	10.3	>10	>10	3.80	>10	>10
**9**	4.15	3.27	4.14	1.80	3.88	6.51
**10**	+	>10	>10	>10	>10	>10
**14**	+	11.0	+	+	>10	+
**16**	+	>10	>10	>10	>10	>10
**17**	>10	>10	>10	>10	>10	>10
**18**	+	+	+	+	+	+
**19**	10.3	>10	+	+	+	+

Values represent IC_{50} in μM. In grey, determined values of possible interest. + indicates an increase of tumor cell line growth in relation to the untreated control test.
